# Topological analysis of the human lymph node reticular network predicts outcome in breast cancer

**DOI:** 10.1002/path.70065

**Published:** 2026-04-20

**Authors:** Amy M Llewellyn, Sharon L D'Costa, Cheun YR Lam, Jasmine A Gore, Veronika Lachina, Daniel J Shewring, Sophie E Acton, Kalnisha Naidoo

**Affiliations:** ^1^ Translational Pathology, Comprehensive Cancer Centre, School of Cancer and Pharmaceutical Sciences King's College London London UK; ^2^ Synnovis, Department of Cellular Pathology a partnership between SYNLAB UK & Ireland, Guy's and St Thomas' NHS Foundation Trust, and King's College Hospital NHS Foundation Trust London UK; ^3^ Stromal Immunology Group, Laboratory for Molecular Cell Biology University College London London UK; ^4^ Department of Cellular Pathology King's College Hospital London UK

**Keywords:** fibroblast reticular cells, breast cancer, axillary lymph nodes, neoadjuvant chemotherapy, immunohistochemistry, conduits

## Abstract

Axillary LNs (ALNs) initiate immune responses in breast cancer (BC) but how and when ALNs become dysfunctional, facilitating metastasis, is unclear. The fibroblastic reticular cell (FRC) network within ALNs provides structural support and mediates immune homeostasis, but we have yet to elucidate whether this network changes during BC progression. An unbiased computational approach was used to quantify features of the immunolabelled FRC network in ALNs derived from patients with BC. Platelet‐derived growth factor receptor β (PDGFRβ) was identified as a robust immunomarker for human FRC and used to quantify how FRC network topology changes during BC progression and after treatment. Formalin‐fixed paraffin‐embedded ALNs (*n* = 331) from 179 patients with BC and 23 benign reactive controls were assessed for FRC network metrics, including lacunarity and branchpoints, alongside de‐identified clinico‐pathological data. These data were then integrated using multivariate, principal component and survival analyses. In node‐negative, post‐neoadjuvant chemotherapy triple‐negative BC, denser FRC networks in uninvolved nodes significantly improved survival (*p* = 0.0365). [Correction added on 09 May 2026, after first online publication: The word ‘treatment‐naïve’ has been corrected to ‘post‐neoadjuvant chemotherapy’ in the preceding sentence.] Conversely, similar changes seen in node‐positive BC significantly worsened survival (*p* = 0.0407), regardless of BC subtype or treatment. In metastatic ALNs, FRC network disruption grew proportionately to axillary tumour burden, and this significantly correlated with poorer outcomes (*p* = 0.043). Interestingly, increased FRC alignment within these metastases significantly improved survival (*p* = 0.0205). This study showed that changes in human ALN FRC network topology predicts BC prognosis. This could improve how we risk stratify patients in future, and provide a new avenue for mechanistic, translational research. © 2026 The Author(s). *The Journal of Pathology* published by John Wiley & Sons Ltd on behalf of The Pathological Society of Great Britain and Ireland.

## Introduction

In early breast cancer (BC), sentinel LN (SLN) status guides management decisions since it is the most significant prognostic factor. However, while axillary LNs (ALNs) are the first site of BC metastasis, they are also highly organised, tightly regulated secondary lymphoid organs capable of generating anti‐tumour responses and modulating fluid homeostasis. Consequently, surgically clearing the axilla can cause arm lymphoedema, but its effects on immune function are unclear [1].

BC treatment now integrates tailored combinations of (neo)adjuvant systemic therapies based on disease stage and hormone receptor status [oestrogen receptor positive (ER‐positive), human epidermal growth factor receptor 2 positive (HER2‐positive) and triple‐negative (TNBC)]. Axillary management has also changed, with the number of ALN clearances (ALNCs) decreasing in favour of the less invasive SLN biopsy (SLNB) and, more recently, targeted axillary dissection [2]. Clinical trials, like Z0011 and AMAROS, catalysed this paradigm shift by challenging the necessity of ALNC in patients with minimal axillary involvement [3, 4]. Specifically, the fact that isolated tumour cells (ITCs; < 200 cells or < 0.2 mm) and micrometastasis (0.2–2 mm) do not impact recurrence or overall disease‐free survival if appropriate systemic therapies are given, supports conserving the axilla [5]. Regardless, precise histopathological examination of every excised ALN is still mandated for accurate prognostication/staging. Furthermore, with neoadjuvant immunotherapy now recommended for TNBC, knowing if the patient's ALN microenvironment will activate or suppress their immune response is clinically relevant [6]. However, our biological understanding of the mechanisms of ALN metastasis, and which factors determine if a patient will mount an effective immune response, remains poorly understood.

Fibroblastic reticular cells (FRCs) are integral to ALN structure and function. These specialised stromal cells secrete and enwrap extracellular matrix (ECM) to form a network of reticular fibres that localise immune cells and transport antigen (reviewed in [7]). Lymph enters the LNs via the subcapsular sinus (SCS), where sinus‐lining cells restrict molecules ≥ 70 kDa from accessing the conduit system [8, 9, 10]. Smaller soluble molecules filter through the FRC‐ensheathed conduit network in a unidirectional, controlled manner, traversing the LN parenchyma to high endothelial venules. Besides providing a structural scaffold, FRCs orchestrate immune cell interactions by producing chemokines such as chemokine ligand 19 (CCL19) and CCL21, which regulate lymphocyte migration. Single cell RNA sequencing (scRNAseq) has elucidated distinct murine FRC subsets with specific roles in maintaining immunological niches [11]. However, the canonical murine FRC marker remains Podoplanin (PDPN). In mice, this membranous glycoprotein plays a central role in immunoregulation and in maintaining/remodelling the conduit network [12, 13, 14].

Conversely, our knowledge of human FRC biology in immunity and cancer is still evolving. Studying human FRC is challenging due to the ethical constraints on accessing lymphoid tissue. In BC, every excised ALN must be formalin fixed and paraffin wax embedded (FFPE) for accurate pathological staging [15], limiting sample availability for research. In addition, FRC comprise 1–5% of human LNs and are difficult to isolate from primary tissue [16, 17]. Furthermore, there is a lack of unique, robust FRC markers (reviewed in [18]). Attempts to isolate human FRCs have traditionally used PDPN to define the population, but PDPN expression is low in human FRCs [19, 20, 21]. Interestingly, recent scRNAseq of reactive (tumour‐free) human cervical LNs showed that platelet‐derived growth factor receptor β (PDGFRβ) is expressed by all LN fibroblasts [22]. Finally, the collagenous reticular network also exists in human LNs, but our understanding of how its structure/composition change in disease remains limited. Histopathologists have historically used the tinctorial stains, reticulin, to visualise this network [23], and Masson's trichrome to highlight mature fibrosis [24], but these methods have not been correlated with clinical data or FRC topology.

One study on freshly resected ALNs containing BC metastases identified four prognostic fibroblast subsets by analysing fibroblast activation protein α1 (FAP), integrin β1, smooth muscle actin (SMA), PDGFRβ and PDPN expression [25]. However, the sample size was small; no benign reactive controls were used; and the spatial distribution of these populations within ALNs was not defined.

Herein, we define robust immunohistochemical (IHC) markers of human FRCs and characterise expression patterns in ALNs derived from patients with BC and reactive, control LNs from patients with benign disease. We comprehensively map and quantify the effect of BC and neoadjuvant chemotherapy (NACT) on FRC network topology in involved and uninvolved ALNs, and link these topological changes to patient survival.

## Materials and methods

### Patient cohort

Archival, de‐identified FFPE ALN samples (*n* = 331) were obtained from 179 patients (Table 1) who underwent a SLNB and/or ALNC for invasive BC at King's College Hospital (2014–2024), through the Breast Cancer Immune, Drug and Gene Study (Research Ethics Committee No: 24/NW/0079). No patients received checkpoint inhibitor therapies (CPIs). For each case, the following de‐identified data were collected: age, molecular subtype, tumour infiltrating lymphocytes (TILs), pathological TNM (pTNM) stage, histological grade, NACT regimen/response, primary tumour size, largest ALN metastasis size and overall survival. Axillary tumour burden (ATB) was calculated by adapting the residual cancer burden (RCB) score formula [26]: tumour volume in the axilla = ((1 − 0.75^LN^) *d*_met_), where LN is the number of positive nodes and *d*_met_ is the largest nodal metastasis diameter. Overall survival was defined as time from diagnosis to death of any cause. We also identified 23 patients with a benign (non‐cancerous) diagnosis who underwent LN biopsy/excision to serve as reactive controls.

**Table 1:** Clinico‐pathological characteristics of the breast cancer cohort.

Clinicopathological characteristic		No (%)	
Age (years)			
< 50		44 (25)	
> 50		135 (75)	
Pathological T stage			
ypT0		55 (31)	
pT1	ypT1	42 (23)	17 (9)
pT2	ypT2	44 (25)	8 (4)
pT3	ypT3	6 (3)	6 (3)
pT4	ypT4	1 (1)	0 (0)
Pathological N stage			
pN0	ypN0	33 (18)	64 (36)
pN1	ypN1	49 (27)	18 (10)
pN2	ypN2	7 (4)	4 (2)
pN3	ypN3	4 (2)	0 (0)
Molecular subtype			
ER positive		25 (14)	
HER2 positive		67 (37)	
Triple negative		87 (49)	
Chemotherapy status			
Post neoadjuvant chemotherapy		87 (49)	
pCR		52 (60)	
RCB1		8 (9)	
RCB2		15 (17)	
RCB3		9 (10)	
Not recorded		3 (4)	
Treatment naïve		92 (51)	
Histological grade			
1		5 (3)	
2		55 (31)	
3		119 (66)	
TILs (HER2 positive and triple negative)			
< 30%		118 (77)	
≥ 30%		36 (23)	
Lymph node procedure			
Sentinel lymph node biopsy		130 (73)	
Up front axillary clearance		49 (27)	
Completion axillary clearance		22	
Number of lymph nodes analysed			
Reactive nodes from benign cases		23	
Uninvolved nodes		227	
Involved nodes		104	
Macrometastasis		71	
Micrometatasis		26	
Isolated tumour cells (post neoadjuvant chemotherapy)		7	

ER, oestrogen receptor; HER2, human epidermal growth factor receptor 2; pCR, pathological complete response; RCB, residual cancer burden; TIL, tumour infiltrating lymphocyte.

Histopathologists (AML/KN) reviewed each case/ALN to confirm disease status and select appropriate blocks for analysis. Where available, an uninvolved and metastatic node were chosen from each patient. In cases containing micrometastasis and macrometastasis, examples of both were selected. In patients who had SLNB followed by ALNC, nodes from both procedures were included. ALNC were not surgically designated by nodal level; thus all uninvolved nodes were considered representative of the entire axillary basin.

### Tinctorial and immunohistochemical staining

Sections were cut at 3 μm thickness. Reticulin and Masson's trichrome staining were performed using standard protocols [24]. Automated IHC protocols were optimised for each antibody on appropriate control tissues prior to cohort testing (Table 2).

**Table 2:** Optimised immunohistochemistry staining protocols for human fibroblast reticular cells.

Stromal cell marker	Antibody clone	Supplier (product code)	Staining platform	Antigen retrieval method	Concentration	Antibody incubation time
Platelet‐derived growth factor receptor beta (PDGFRβ)	RM303 (recombinant rabbit monoclonal)	Invitrogen, Fisher Scientific UK, Loughborough, UK (MA5‐33050)	Leica Bond III Autostainer	Heat induced, 30 min, buffer pH 9	1 in 100	30 min
Podoplanin (PDPN)	D240 (mouse monoclonal)	Cell Marque, Roche Diagnostics, Burgess Hill, West Sussex, UK (322 M‐10)	Ventana Benchmark Ultra	Heat induced, 15 min, buffer pH 7.5	Ready to use kit, prediluted	20 min
Smooth muscle actin (SMA)	1A4 (mouse monoclonal)	Cell Marque (202 M‐90)	Ventana Benchmark Ultra	Heat induced, 15 min, buffer pH 7.5	Ready to use kit, prediluted	20 min
Integrin beta 1	EP1041Y (rabbit monoclonal)	Abcam, Cambridge, UK (ab52971)	Leica Bond III Autostainer	Enzymatic using proteinase K, 10 min	1 in 300	30 min

### Region of interest (ROI) selection

Whole‐slide images (WSIs) were acquired using the Glissando Desktop Scanner (Objective Imaging, Cambridge, UK) and viewed using QuPath (v0.5.1) [27]. For each PDGFRβ‐stained LN section, eight ROIs (250,000 μm^2^) were manually selected (×200 magnification). In uninvolved nodes, ROIs were chosen from T‐cell zones (paracortex and interfollicular regions), avoiding germinal centres (GCs). In involved ALNs, eight ROIs were selected within metastatic tumours. Nodes bearing only ITCs, micrometastases or small macrometastases were excluded if the tumour was smaller than the standardised ROI. In partially replaced positive nodes, an additional eight ROIs were selected from residual lymphoid tissue at least 500 μm away from the metastatic tumour. In cases with NACT‐induced fibrosis, four additional ROIs were selected from fibrotic areas. In LNs smaller than eight ROIs, the maximum feasible number of ROIs was selected.

### Image analysis

ROIs were analysed using the Workflow of Matrix BioLogy Informatics (TWOMBLI) ImageJ plugin to quantify spatial FRC network metrics [28, 29]. Optimised parameters applied to all images were: contrast saturation = 0.35, minimum line width = 5, maximum line width = 20, minimum branch length = 10 and maximum display high‐density matrix intensity (HDMI) = 237. TWOMBLI derived outputs were: lacunarity (size and frequency of network gaps; HDMI; ROI proportion covered by network); hyphal growth unit (HGU; number of endpoints per unit length); fibre alignment, length, width; number of endpoints and number of branchpoints (supplementary material, Figure S1). Cells were counted using QuPath [27]. ROIs from metastatic ALNs and NACT‐induced fibrosis were additionally analysed using CurveAlign (v5.0) [30].

### Statistical analyses

For each LN, the median across eight ROIs was calculated for all parameters to minimise outlier impact. Data were analysed in RStudio (version 2024.09.1+394). Associations between TWOMBLI derived matrix features and clinical variables were assessed using multivariate linear regression. Unmatched, categorical subgroups were compared using the Kruskal–Wallis test in GraphPad Prism (version 10.4.1). Principal component analysis (PCA) included both TWOMBLI and clinical parameters – apart from HGU, fibre length and width, which were derived from other parameters. Survival analysis was conducted using the Kaplan–Meier method; group comparisons were performed using the Gehan–Breslow–Wilcoxon method.

## Results

### Optimised human FRC subset immunomarkers

We analysed 331 FFPE ALN samples from 179 patients with BC encompassing all three molecular subtypes, different pTNM stages and responses to NACT (Table 1). Twenty‐three tumour‐free LNs from patients with benign disease served as reactive controls. Given the paucity of robust human FRC immunostains, we optimised five previously reported FRC markers [31] and compared staining patterns across serial sections to reticulin, which highlights the collagen backbone of lymphoreticular organs [23].

**Figure 1:**
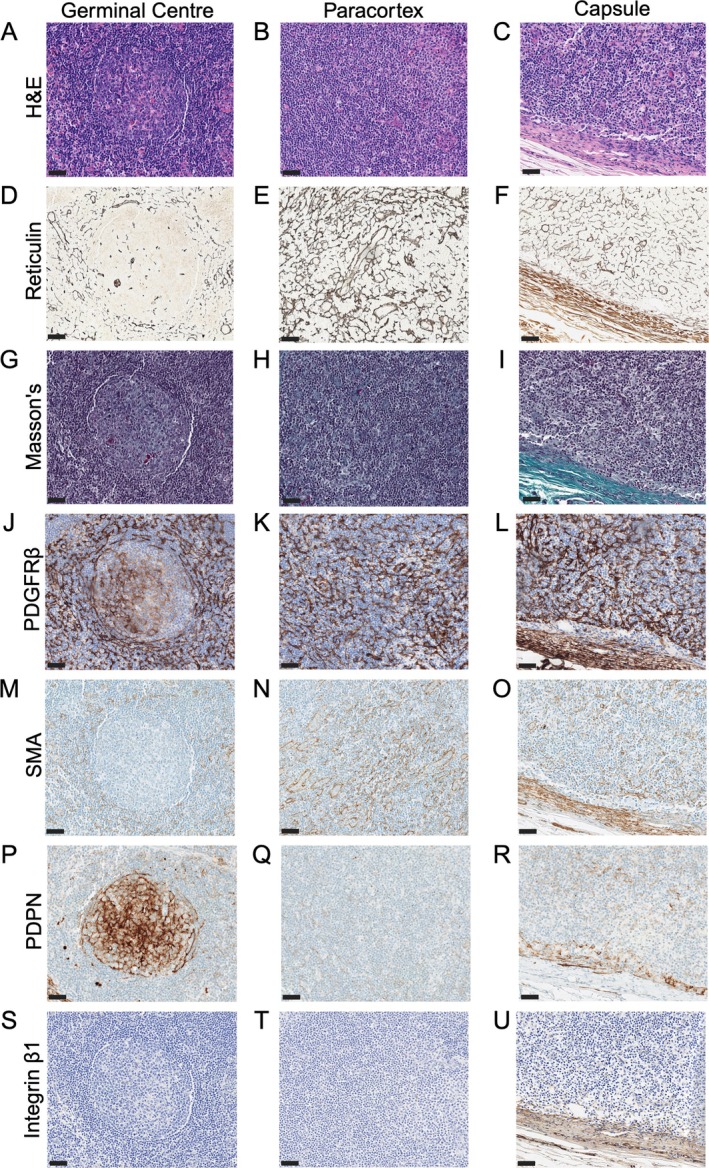
Optimised immunohistochemical (IHC) markers of human fibroblastic reticular cell (FRC) subsets. Representative photomicrographs showing optimised IHC staining of reactive human LN tissue from patients with benign disease (*n* = 23 nodes; scale bars, 50 μm; all images at ×200 magnification). (A–C) The reticular network was not visible by H&E staining in (A) the germinal centres, (B) the paracortex (T‐cell zone) or (C) the capsule. (D–F) Reticulin staining highlighted the reticular collagen network weakly in (D) germinal centres but strongly in (E) the paracortex. (F) The capsule demonstrated a distinct reticulin staining pattern, characterised by thicker, wavier fibres. (G–I) Masson's trichrome did not stain the reticular collagen network in (G) germinal centres or (H) the T‐cell zone, but stained (I) the mature, capsular collagen blue. (K, L) Platelet‐derived growth factor receptor β (PDGFRβ) stained FRCs in (K) the paracortex, with weak follicular dendritic cell (FDC) staining in (J) germinal centres and strong fibroblast staining in (L) the capsule. This pattern mirrored reticulin staining (panels D–F). (M–O) Smooth muscle actin (SMA) was absent in (M) germinal centres, but stained FRCs in (N) the paracortex and (O) the capsule. (P–R) Podoplanin (PDPN) stained FDCs in (P) germinal centres and (R) lymphatic endothelial cells, but did not label FRCs in (Q) the T‐cell zone. (S–U) Integrin β1 staining was restricted to (U) mature blood vessels and the capsule, with no staining of (S) FDCs or (T) FRCs. Created with BioRender.com; Naidoo, K. (2026) https://BioRender.com/m3c9baa.

PDGFRβ mirrored reticulin staining, proving the most robust pan‐FRC marker. It showed consistently strong staining in T‐cell zones, weak follicular dendritic cell (FDC) staining in GCs and strong capsular staining (Figure 1). SMA strongly marked FRCs in the paracortex and capsule, with weak staining in parafollicular zones; it was absent in GC. PDPN strongly stained lymphatic endothelial cells (LECs) and moderately labelled GC FDCs, but did not highlight T‐cell zone FRCs. Integrin β1 was restricted to mature blood vessels and the capsule. FAP staining was technically challenging – the high temperatures required to retrieve antigen and achieve a signal damaged the tissue. Therefore, FAP was not pursued further.

### Quantitative metrics capture FRC network architecture in human LNs

**Figure 2:**
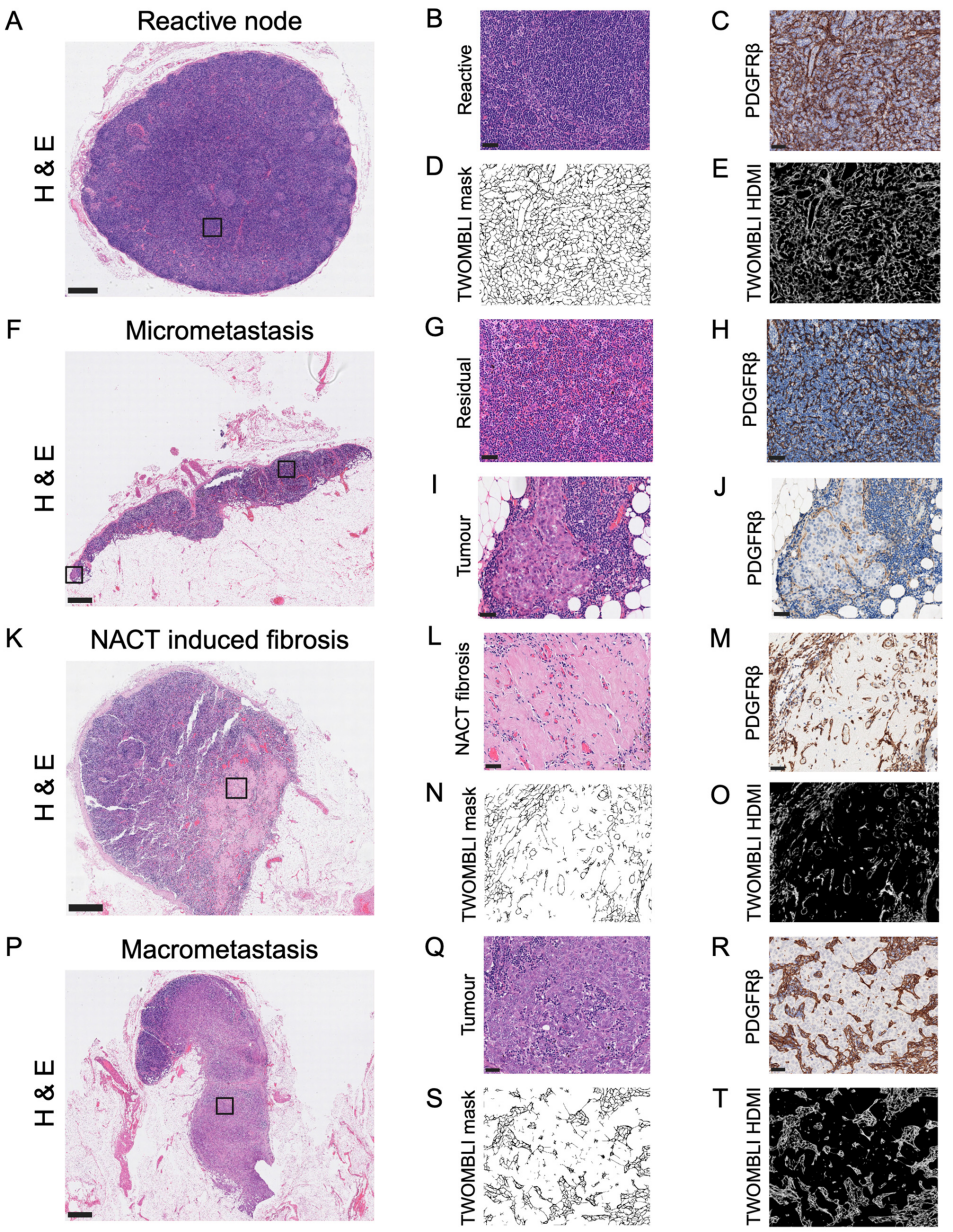
Quantitative metrics capture fibroblastic reticular cell (FRC) network architecture in human LNs. Regions of interest (ROIs) were selected for TWOMBLI analyses from (A) reactive benign control (*n* = 23), (F) uninvolved (*n* = 214), (K) tumour‐infiltrated (*n* = 75) and (P) fibrotic (*n* = 16) nodes, as well as residual lymphoid tissue in (F) metastatic nodes (*n* = 72) (bird's eye view images, scale bars, 1 mm). (B, G, I, L, Q) High‐magnification images of boxed areas, each an example of analysed ROIs. (C, H, M, R) Platelet‐derived growth factor receptor β (PDGFRβ) marked a dense FRC network in (C) benign control and uninvolved nodes, as well as in (H) residual lymphoid tissue > 500 μm from small tumour deposits. (J, R) In metastatic nodes, the (J) small and (R) large tumour deposits showed a disrupted, stretched FRC network. (M) In LNs where tumour had been eradicated by neoadjuvant chemotherapy, PDGFRβ highlighted only a few remaining, aligned FRCs within residual fibrosis. (D, N, S) TWOMBLI masks were generated from each PDGFRβ‐stained ROI to extract FRC/matrix features. This highlighted increased lacunarity in axillary LNs (ALNs) containing (S) large tumour deposits compared with (D) reactive nodes. (E, O, T) TWOMBLI masks were then processed and thresholded to calculate high‐density matrix intensity (HDMI). This showed reduced HDMI in (O) post‐neoadjuvant chemotherapy (NACT) fibrosis compared with (E) reactive nodes. All images apart from bird's eye view are shown at ×200 magnification. Scale bars, 50 μm. Created with BioRender.com; Naidoo, K. (2026) https://BioRender.com/3i4ypum.

Representative ROIs were chosen from PDGFRβ‐stained T‐cell zones within benign, reactive control nodes, uninvolved nodes from patients with BC, residual lymphoid tissue from nodes with smaller metastases, areas of NACT‐induced fibrosis and areas of metastatic tumour (Figure 2). Since subtle alterations in FRC topology could potentially alter function, we needed a precise, unbiased approach to quantify changes in response to BC and/or NACT. TWOMBLI is an image analysis pipeline designed to quantify multiple ECM parameters in tissue [29]. Since FRCs enwrap the reticulin network, we repurposed this pipeline to analyse FRC network parameters [7]. TWOMBLI analysis showed that the FRC network in benign, reactive control LNs (clinico‐pathological characteristics shown in supplementary material, Table S1) has low lacunarity [median = 6, interquartile range (IQR) = 5–7.8], moderate branching (median branchpoints normalised by fibre length = 0.066, IQR = 0.059–0.068), consistent FRC size (median width = 1.5 μm, IQR = 1.3–2.0; median fibre length = 24 μm, IQR 23–34) and is poorly aligned (median alignment = 0.053, IQR = 0.04–0.077). In this small cohort, these network features did not correlate significantly with age (supplementary material, Table S2).

### Treatment‐naïve TNBC and NACT remodel the FRC network in uninvolved ALNs

Multivariate linear analysis of each TWOMBLI output from uninvolved nodes from patients with BC against the clinico‐pathological variables (Table 1) revealed statistically significant associations (supplementary material, Table S3). The number of endpoints in the reticular network yielded the highest R score (0.127, *p* = 0.000007), with molecular subtype (*p* = 0.0006) and ATB (*p* = 0.01) as significant predictors. Fibre width (*p* = 0.00006), number of branchpoints (*p* = 0.00009), lacunarity (*p* = 0.0001), fibre length (*p* = 0.00007), HDMI (*p* = 0.001) and HGU (*p* = 0.05) were significantly predicted by NACT exposure.

**Figure 3:**
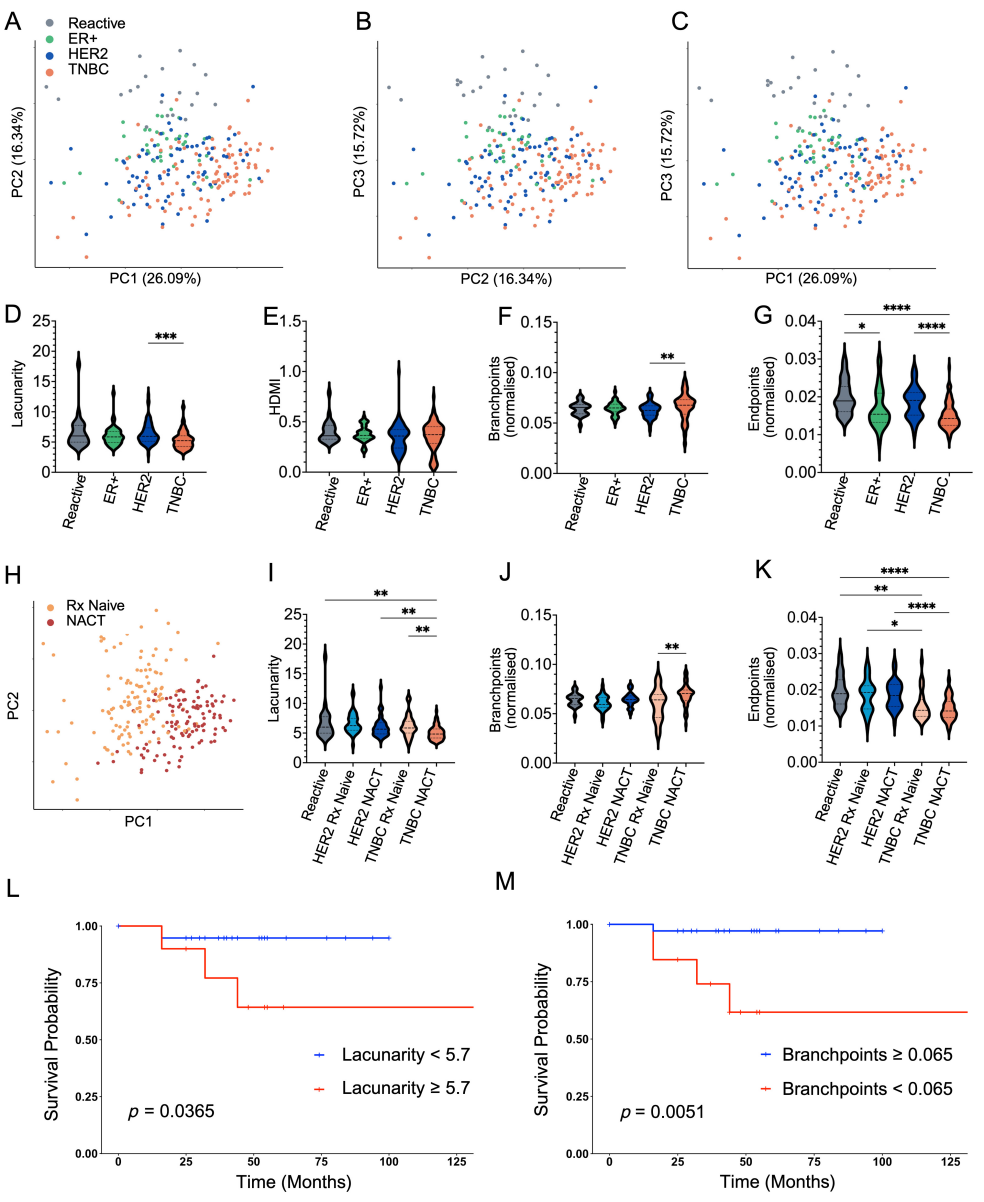
Treatment‐naïve triple negative breast cancer (TNBC) and neoadjuvant chemotherapy (NACT) remodel the fibroblastic reticular cell (FRC) network in uninvolved axillary LNs (ALNs). (A–C) Principal component analysis (PCA) was performed to integrate the TWOMBLI derived FRC network features and clinico‐pathological variables obtained from benign control nodes (*n* = 23) and uninvolved ALNs (oestrogen receptor‐positive (ER+): *n* = 40; human epidermal growth factor receptor 2 (HER2): *n* = 78; and TNBC: *n* = 96). PC1 accounted for 26.09% of the total variance, predominantly driven by lacunarity (loading = −0.506), followed by branchpoints (loading = 0.478) and NACT (loading = 0.403). PC2 and PC3 contributed 16.34% and 15.72%, respectively, to variance (see also supplementary material, Figure S2 and Table S3). Benign control nodes always clustered separately to uninvolved ALNs from patients with breast cancer (BC). (D–G) Violin plots showing molecular subtype‐specific differences in (D) FRC network lacunarity, (E) high‐density matrix intensity (HDMI), (F) number of branchpoints and (G) number of endpoints in uninvolved ALNs (see also supplementary material, Figure S2). (H) PCA showing the effect of NACT on uninvolved nodes from all BC molecular subtypes (treatment naïve: *n* = 126 ALNs; NACT: *n* = 111 ALNs). (I–K) Violin plots illustrating the impact of NACT on (I) FRC network lacunarity, (J) number of branchpoints and (K) number of endpoints in uninvolved ALNs, stratified by BC molecular subtype (reactive: *n* = 23 nodes, HER2 treatment naïve: *n* = 39 nodes, HER2 post‐NACT: *n* = 39 nodes, TNBC treatment naïve: *n* = 29 nodes, TNBC post‐NACT: *n* = 67 nodes). (L, M) Kaplan–Meier survival analysis of patients with TNBC with ypN0 disease (i.e. no residual disease in the axilla post‐NACT) shows that patients with uninvolved ALNs that show a lacunarity ≥ 5.7 (*p* = 0.0365) or branchpoints < 0.065 (*p* = 0.0051) have a significantly worse overall survival (*n* = 49 nodes; Gehan–Breslow–Wilcoxon method). All violin plots show median with interquartile range, with minimum and maximum, and were analysed with the Kruskal–Wallis test. For PCA and violin plots, each data point represents the median of the results from eight regions of interest (ROIs) from each node. *p ≤ 0.05, **p ≤ 0.01, ***p ≤ 0.001, ****p ≤ 0.0001. Created with BioRender.com; Naidoo, K. (2026) https://BioRender.com/zxjkil3.

To assess network parameters alongside clinical variables in a more integrative manner, we performed PCA on the combined dataset (Figure 3A–C). PC1 accounted for 26.09% of the total variance, predominantly driven by lacunarity (loading = −0.506), then branchpoints (loading = 0.478) and NACT (loading = 0.403) (supplementary material, Table S4 and Figure S2). Thus, PC1 captures variability driven by both network structural features and clinical context. Other principal components indicated complex relationships across topological and clinical parameters (supplementary material, Table S4). Strikingly, benign control nodes always clustered separately from uninvolved ALNs from patients with BC, suggesting cancer‐specific changes to ALN structure. There were no significant associations between TILs, RCB score, clinical or topological parameters in HER2‐positive and triple‐negative BC (supplementary material, Figure S3 and Tables S5–S7).

Furthermore, we observed topological alterations linked to BC subtype. Stratification of TWOMBLI results from patients who were treatment naïve and those who were NACT exposed by molecular subtype revealed that FRC networks in uninvolved TNBC nodes had significantly reduced lacunarity (*p* < 0.001; Figure 3D), unchanged HDMI (Figure 3E), increased branchpoints (*p* = 0.0048; Figure 3F) and fewer endpoints (*p* < 0.0001; Figure 3G) than HER2‐positive and reactive nodes, independent of treatment. Visualisation of PCA distribution by NACT exposure also clearly segregated patients who were treatment naïve from those who were NACT exposed (Figure 3H). Subsequent PCA faceting by both molecular subtype and NACT exposure distinctly separated uninvolved and reactive control nodes (supplementary material, Figure S4). This separation was more pronounced in TNBC than HER2‐positive disease, and was accentuated in NACT‐exposed nodes, indicating that both tumour subtype and therapy exposure contribute to network remodelling. Further stratification of individual TWOMBLI parameters according to subtype and NACT exposure confirmed these patterns. FRC networks from patients with NACT‐exposed TNBC displayed significantly decreased lacunarity (*p* < 0.01; Figure 3I), increased branchpoints (*p* < 0.01; Figure 3J) and decreased endpoints (*p* < 0.0001; Figure 3K; supplementary material, Figure S4).

Collectively, this indicates that in patients with TNBC who were treatment naïve, the FRC network in uninvolved ALNs is more compact and highly branched. Interestingly, NACT induces comparable alterations throughout the ALN basin. Notably, Kaplan–Meier analysis of patients with TNBC with no axillary nodal involvement following NACT (ypN0) demonstrated that the presence of a denser FRC network, characterised by lower lacunarity (*p* = 0.0365, Figure 3L) and more branchpoints (*p* = 0.0051, Figure 3M), significantly improved survival.

### Metastatic tumour deposits disrupt the FRC network in adjacent residual lymphoid tissue

To test how BC affects the FRC network within a single node, we repeated our analysis including ROI of residual lymphoid tissue in involved nodes across all molecular subtypes (treatment naïve and post NACT). Multivariate analysis revealed lacunarity as the parameter most significantly influenced by clinical factors (R score 0.157, *p* = 2 × 10^−9^) with metastasis size, NACT exposure and molecular subtype as the most significant predictors (supplementary material, Table S8). Direct comparison of TWOMBLI outputs from treatment‐naïve and post‐NACT nodes confirmed the presence of a denser, more branched network in uninvolved nodes post‐NACT (supplementary material, Figure S5). Furthermore, stratification by molecular subtype confirmed the significant effect of TNBC on FRC topology.

**Figure 4:**
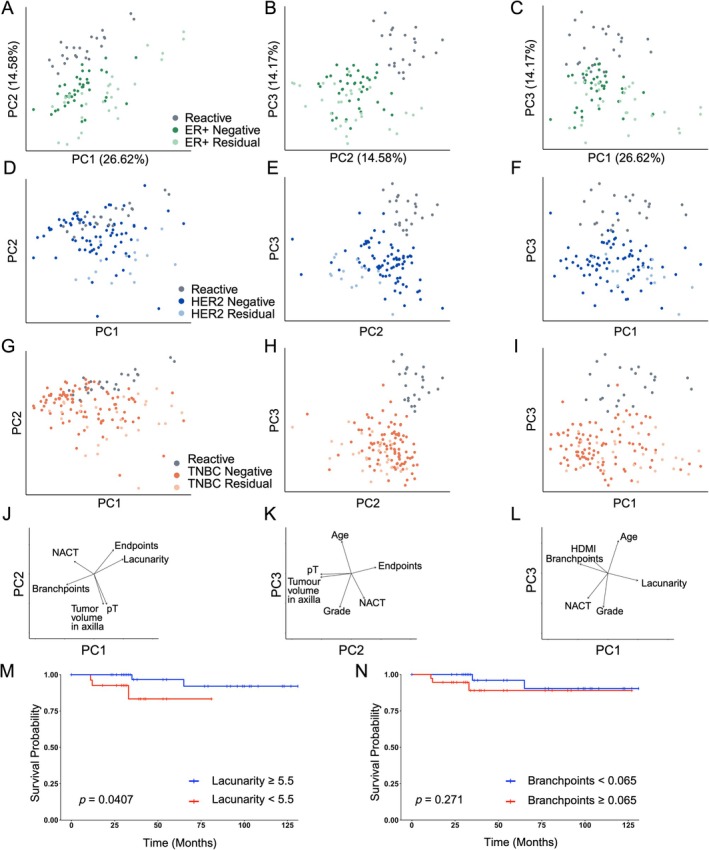
Metastatic tumour deposits disrupt the fibroblastic reticular cell (FRC) network in adjacent residual lymphoid tissue. Principal component analysis (PCA), stratified by breast cancer (BC) molecular subtype, was performed to integrate TWOMBLI‐derived FRC network features and clinico‐pathological variables from benign control nodes (*n* = 23), uninvolved axillary LNs (ALNs) and residual LN tissue from involved ALNs. PC1, PC2 and PC3 contribute 26.62%, 14.85% and 11.47% of the variance, respectively (see also supplementary material, Figures S2, S5 and Table S9). Each data point represents the median of eight regions of interest (ROIs) per node. Once again, benign control nodes clustered distinctly from nodes derived from patients with BC. (A–C) ALNs from patients with oestrogen receptor (ER)‐positive BC (ER+ uninvolved: *n* = 40 nodes, ER+ residual: *n* = 29 nodes). (D–F) ALNs from patients with HER2‐positive disease (HER2 uninvolved: *n* = 78 nodes, HER2 residual: *n* = 15 nodes). (G–I) ALNs from patients with triple negative BC (TNBC uninvolved: *n* = 96 nodes, TNBC residual: *n* = 28 nodes). (J–L) Loading plots illustrating the contribution of the top six individual features to the first three principal components. (M, N) Kaplan–Meier survival analysis of patients with pN1 or ypN1 disease, of any molecular subtype, showed that patients with uninvolved ALNs with a lacunarity < 5.5 have a significantly worse overall survival (*p* = 0.0407) but that branchpoints ≥ 0.065 (*p* = 0.271) is not a significant predictor (*n* = 82 nodes; Gehan–Breslow–Wilcoxon method). Created with BioRender.com; Naidoo, K. (2026) https://BioRender.com/d499dm2.

PCA again demonstrated that across all subtypes, ALNs derived from patients with BC clustered distinctly from reactive, benign control nodes (Figure 4A–I). Moreover, within each molecular subtype, metastatic ALNs containing residual lymphoid tissue clustered separately to uninvolved nodes. This indicates that the effects of BC on the stromal network are more pronounced in the residual lymphoid tissue adjacent to tumour deposits in metastatic ALNs. Interestingly, lacunarity had the highest loading on PC1 (0.410), accounting for 26.62% of data variance (supplementary material, Table S9 and Figure S2). This aligned with the multivariate analysis (supplementary material, Table S8). Furthermore, PC1, PC2 and PC3 all contributed to meaningful separation of the data (Figure 4J–L). Overall, these results confirm that TNBC and NACT induce a more compact FRC network in the residual lymphoid tissue of involved nodes.

We specifically investigated the impact of tumour metastasis in ALNs on the FRC topology of uninvolved nodes within the same chain and linked these data with patient survival. In patients with metastatic BC involving one to three ALNs (treatment naïve and/or post NACT), a more compact FRC network, indicated by reduced lacunarity, in the uninvolved nodes was significantly associated with poorer overall survival, independent of treatment status (*p* = 0.0407; Figure 4M). In this cohort, branchpoints did not correlate with survival (*p* = 0.271; Figure 4N).

### Chemotherapy‐induced fibrosis replaces reticular network architecture

**Figure 5:**
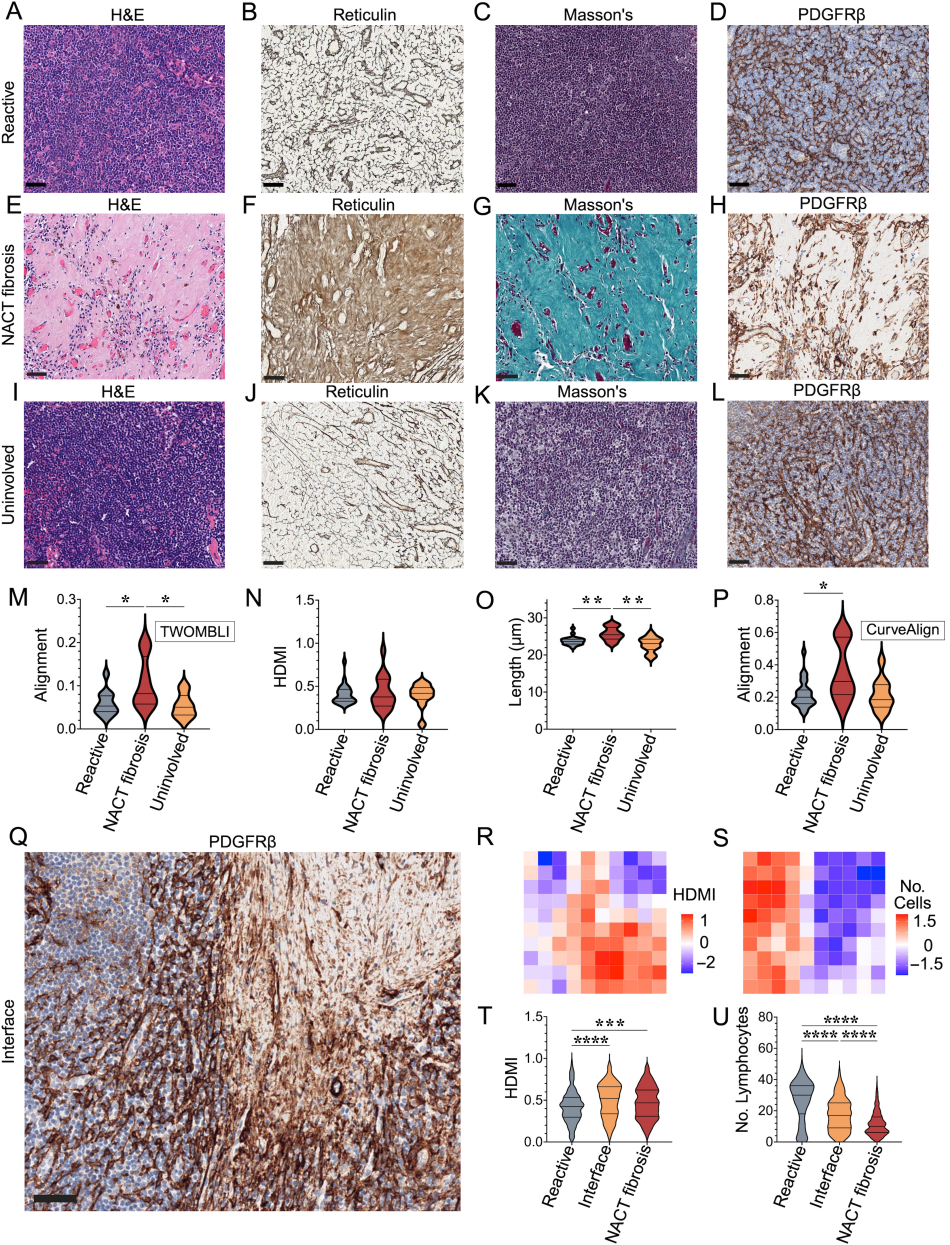
Chemotherapy‐induced fibrosis replaces reticular network architecture. (A–D) Representative photomicrographs of benign control node regions of interest (ROIs) (*n* = 23). The reticular network was not visible by (A) H&E staining, but was highlighted by (B) reticulin staining. (C) Masson's Trichrome showed no mature collagen within the node. (D) Platelet‐derived growth factor receptor β (PDGFRβ) stained the delicate fibroblastic reticular cell (FRC) network. (E–H) Representative photomicrographs of ROIs from areas of chemotherapy‐induced fibrosis (*n* = 16 nodes). (E) H&E staining showed dense eosinophilic collagen with sparse lymphocytes. (F) Reticulin staining showed complete replacement of the normal reticular network by thicker argyrophilic fibres. (G) Masson's trichrome staining demonstrated abundant mature (blue) collagen. (H) PDGFRβ immunohistochemistry revealed complete destruction of the FRC network with few remaining FRCs. (I–L) Representative photomicrographs of ROIs from areas of uninvolved axillary LNs (ALNs) that were removed from the same patients as those containing neoadjuvant chemotherapy (NACT)‐induced fibrosis (*n* = 16 nodes). (I) H&E staining showed only lymphoid tissue, with no areas of fibrosis and no evidence of tumour deposits. (J) Reticulin staining showed a normal reticular network. (K) Masson's trichrome stain showed no mature collagen. (L) PDGFRβ immunostaining revealed a preserved FRC network. (M–P) Violin plots comparing (M, P) FRC alignment, (N) high‐density matrix intensity (HDMI) and (O) length across reactive LNs (*n* = 23) to areas of chemotherapy‐induced fibrosis and matched uninvolved (negative) ALNs from the same patient (*n* = 16). FRC alignment was calculated using both (M) TWOMBLI software and (P) CurveAlign software. Each data point represents a median of the results from four ROIs from each node. (Q) Representative photomicrograph of a PDGFRβ‐stained ROI showing the interface between chemotherapy‐induced fibrosis and the residual FRC network (*n* = 13 nodes). (R, S) Examples of heatmaps of TWOMBLI analyses generated from ROIs in (Q) illustrating the gradual increase in (R) HDMI and (S) decrease in lymphocyte cell number across the transition from an area of residual lymphoid tissue to an area of NACT‐induced fibrosis. (T, U) Violin plots of the aggregated heatmap data comparing (T) HDMI and (U) number of lymphocytes across areas of residual LNs, interface and areas of NACT‐induced fibrosis (*n* = 13 nodes). All images shown at ×200 magnification, scale bars, 50 μm. All violin plots show median with interquartile range, with minimum and maximum and were analysed with the Kruskal–Wallis test. *p ≤ 0.05, **p ≤ 0.01, ***p ≤ 0.001, ****p ≤ 0.0001. Created with BioRender.com; Naidoo, K. (2026) https://BioRender.com/jv1uq9e.

The FRC/reticulin network spans the entire reactive, benign control node (Figure 5A–D). In contrast, NACT induces wedge‐shaped fibrosis where tumour cells are replaced by dense eosinophilic collagen and angiogenic vessels (Figure 5E). Clinically, this indicates treatment response [32]. However, the nature and effect of this fibrosis on the FRC network has not been characterised previously. Reticulin staining of these areas revealed abundant mature collagen composed of thicker, wavy fibres that replaced the normal reticular network (Figure 5F). These fibres resembled those within the LN capsule, and stained blue with Masson's trichrome (Figure 5G). PDGFRβ immunostaining demonstrated complete network disruption with fewer, scattered FRCs (Figure 5H). Notably, these changes were not visible in matched uninvolved nodes (Figure 5I–L) from post‐NACT BC patients. TWOMBLI analysis revealed increased FRC alignment (*p* = 0.0210; Figure 5M), non‐significant changes in HDMI (Figure 5N) and a significant reduction in FRC length (*p* = 0.002; Figure 5O) in this fibrosis. However, TWOMBLI is not optimised for assessing alignment, particularly when the network has been destroyed. Therefore, the same ROIs were analysed using CurveAlign [30], which trended towards increased FRC alignment in post‐NACT fibrotic regions compared to matched, uninvolved ALNs (*p* = 0.09; Figure 5P).

PDGFRβ staining at the interface between NACT‐induced fibrosis and the remaining FRC network showed a transition from destroyed to disrupted to intact FRC network architecture (Figure 5Q). For high‐resolution quantification, each interface ROI was subdivided into 100 tiles. These were individually analysed using TWOMBLI, and reconstructed into heatmaps to visually represent the gradual changes in network parameters across the residual node, interface and fibrotic area (Figure 5R,S). Aggregated heatmap data showed the highest average HDMI values in the interface region (*p* ≤ 0.0001; Figure 5T), and a gradual decrease in lymphocyte number through this area of transition (*p* ≤ 0.0001; Figure 5U).

### FRC network topology in metastatic ALNs correlates with ATB and clinical outcome

**Figure 6:**
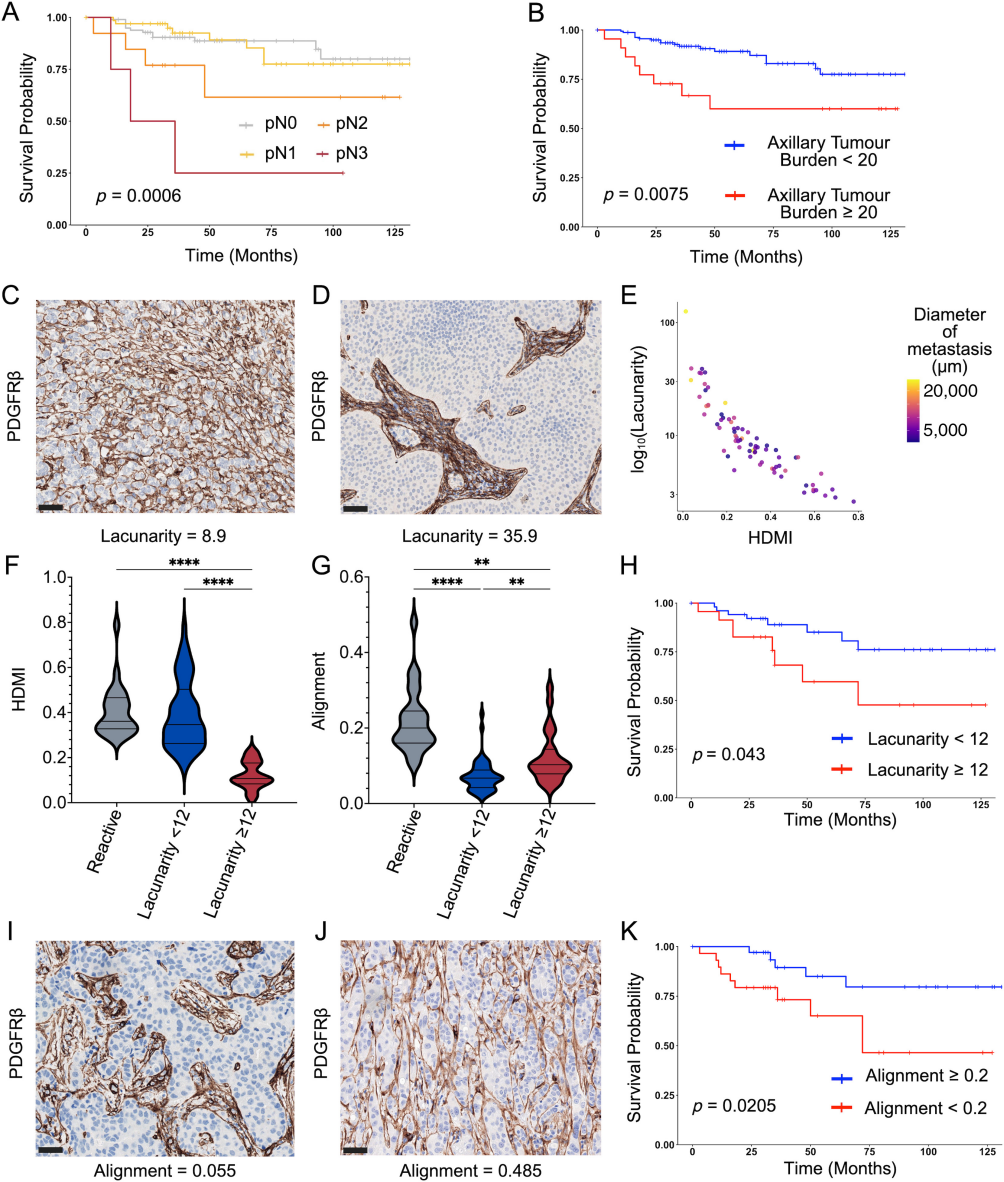
Fibroblastic reticular cell (FRC) network topology correlates with axillary tumour burden (ATB) and clinical outcome in metastatic axillary LNs (ALNs). (A, B) Kaplan–Meier survival curves showing reduced survival in patients with higher nodal stage (A; *p* = 0.0006) and axillary tumour volume ≥ 20 (B; *p* = 0.0075; *n* = 179 patients; see also supplementary material, Figure S4). (C, D) Representative photomicrographs showing regions of interest (ROIs) of macrometastases with different lacunarities in ALNs stained with platelet‐derived growth factor receptor β (PDGFRβ). (E) Scatterplot showing the inverse correlation between TWOMBLI‐derived lacunarity and high‐density matrix intensity (HDMI) in metastatic ALNs. Data point colour reflects metastatic tumour diameter (μm); larger metastases are associated with higher lacunarity and reduced FRC network density (*n* = 75 nodes). (F, G) Violin plots (median with interquartile range, with minimum and maximum) comparing (F) FRC network HDMI and (G) alignment in involved ALNs, stratified according to lacunarity (involved ALNs with lacunarity < 12: *n* = 52; involved ALNs with lacunarity ≥ 12: *n* = 23) with benign control nodes (*n* = 23; Kruskal–Wallis test; **p ≤ 0.01, ****p ≤ 0.0001). (H) Kaplan–Meier survival analysis, stratified by lacunarity in metastatic nodes, showed that patients with positive nodes showing high lacunarity have significantly poorer survival compared with those with low lacunarity (*p* = 0.043; *n* = 64 patients). (I, J) Representative photomicrographs showing ROIs of (I) a macrometastasis with an alignment of 0.055 calculated using CurveAlign software), as opposed to (J) a macrometastasis with an alignment of 0.4, in ALN sstained with PDGFRβ. (K) Kaplan–Meier survival analysis, stratified by alignment in metastatic nodes, showed that patients with involved nodes showing an FRC alignment < 0.2 (calculated using CurveAlign software) have significantly poorer survival compared with those with higher FRC alignment (*p* = 0.0205; *n* = 64 patients). All images shown at ×200 magnification. Scale bar, 50 μm. For panels (E–G), each data point represents a median of the results from eight ROIs from each node. Kaplan–Meier significance calculated using Gehan–Breslow–Wilcoxon method. Created with BioRender.com; Naidoo, K. (2026) https://BioRender.com/h9nrbcr.

As expected, survival analysis of this cohort demonstrated a poorer prognosis for patients with higher nodal stage (Figure 6A), TNBC, advanced tumour stage and RCB 3 (supplementary material, Figure S6). However, traditional nodal stage incorporates the number of positive nodes but not the metastatic deposit size, which we have shown to be pertinent to network topology. Clinically, ATB is routinely quantified in post‐NACT, but not treatment‐naïve, samples [26]. To compare these two patient cohorts, we adapted the RCB score formula to calculate ATB [26]. ATB stratification revealed that patients with a tumour volume ≥ 20 had significantly worse outcomes (*p* = 0.0075; Figure 6B). As tumour cells invade ALNs, they progressively distort the FRC network, quantified as a significant increase in lacunarity correlated with a decrease in HDMI (Figure 6C–G). The degree of network distortion correlated with the metastatic deposit size, rather than molecular subtype or NACT exposure (Figure 6C–G). Notably, large tumour nests resulting in high network lacunarity (≥ 12) were associated with significantly poorer survival (*p* = 0.043, Figure 6H). Surprisingly, in all patients with BC, irrespective of subtype and treatment status, increased FRC alignment in metastatic deposits correlated with improved survival (*p* = 0.0205, Figure 6I–K).

## Discussion

By optimising robust human FRC immunomarkers, we have shown how different FRC subsets localise within ALNs derived from patients with BCs. We confirmed PDGFRβ as a pan‐FRC marker that highlights FRCs in all microanatomical ALN compartments, where different immune populations reside/traffic in health and disease [22]. Interestingly, unlike murine Pdpn which stains FRCs in T‐cell zones, human PDPN is expressed predominantly by GC FDCs and LECs [14]. Furthermore, SMA expression is more prominent in the human LN paracortex than in mice, where it weakly stains GCs, increasing upon activation. These differences could be either an inherent variation between species, or age related, since most mouse models use young, immunologically naïve mice, whereas most excised human nodes will have undergone repeated cycles of antigen exposure, immune activation and resolution. Moreover, since PDPN is observed in FRCs within tertiary lymphoid structures in non‐small cell lung cancer, expression may be context dependent [33]. Regardless, this biological variability could compromise clinical translational, and therefore warrants further investigation.

Because FRCs enwrap collagen in LNs [34], we repurposed TWOMBLI as an unbiased, quantitative tool to map the human nodal FRC network [29]. By integrating TWOMBLI derived parameters with clinical data through multivariate analysis, we could visualise FRC network changes in a translationally relevant context. Importantly, our approach is transferable to other disease processes and can be automated for use in routine diagnostic practice or clinical trials.

The FRC network was denser and more highly branched in treatment‐naïve TNBC, an aggressive subtype. We postulate that this denser FRC network may restrict lymph flow through the ALN parenchyma, shunting fluid through the SCS into efferent vessels. This could have two clinically relevant effects. Firstly, subcapsular pooling of fluid may facilitate cancer cell extravasation and metastatic outgrowth [35]. Secondly, decreasing antigen flow through the ALN parenchyma could disrupt immune cell interactions essential to mounting an effective anti‐tumour immune response [10]. These changes trended towards a poorer prognosis in our treatment‐naïve TNBC cohort, but did not reach statistical significance. This probably reflects the difficulty in obtaining treatment‐naïve samples, since most patients receive NACT [36].

Interestingly, these topological changes were evident in patients without nodal metastasis and in uninvolved ALNs from patients with metastases elsewhere in the axillary chain. Previous mouse studies have shown that factors secreted by primary melanoma travel to tumour‐draining LNs, promoting FRC proliferation and a shift toward a cancer‐associated fibroblast (CAF)‐like phenotype [37]. Thus, soluble factors from the primary breast tumour could induce the structural changes seen in pN0 patients. Conversely, the presence of similar topological changes in uninvolved nodes from patients with metastasis in other ALNs (pN1/ypN1) could be due to soluble factors travelling between ALNs. This makes it difficult to unpick precisely what is occurring in patients with NACT exposure, since these patients could have had ALN metastases which responded to preoperative treatment. Thus, inter‐nodal communication cannot be entirely excluded in ypN0 cases. This confounding effect is not seen once metastases are established in the axillary chain, however. In uninvolved nodes from patients with node‐positive disease (all subtypes), decreased lacunarity was significantly associated with poorer prognosis, regardless of treatment status. This is clinically important as deciding on the extent of axillary surgery in this node‐positive cohort is often challenging [3]. Therefore, any biomarker that can pinpoint when SLN immunity is compromised would be invaluable.

TNBC is the most immunogenic BC subtype. Some TNBC tumours are lymphocyte predominant and the ALNs of patients with TNBC often contain more GCs [38, 39]. Despite this, CPI are only effective when combined with NACT [40], and even then, only 60% of patients with TNBC achieve a complete pathological response (pCR) [41]. We have shown that the FRC network of uninvolved ALNs is significantly denser and more highly branched post‐NACT in patients with TNBC. Intriguingly, in contrast to patients who are treatment naïve, this change in FRC topology significantly improved survival in patients with TNBC with no residual axillary metastasis. This suggests that the same alterations in fluid flow, with subcapsular shunting, are beneficial in this cohort, potentially by facilitating drug‐induced killing of subcapsular micrometastases.

In metastatic ALNs, chemotherapy concurrently eradicates large tumour deposits and the FRC network, inducing dense, mature fibrosis containing a few, highly aligned FRCs and scattered lymphocytes. These structural changes could impede immune cell trafficking/activation, and investigating this further might help to resolve whether patients with a pCR post‐NACT should have a targeted axillary dissection [42]. Moreover, since CPI + NACT elicits mixed responses, understanding the many factors that influence treatment response is crucial. We aim to confirm and further characterise this in a larger cohort of patients in future research, and would encourage others to do the same, especially in clinical trials evaluating new therapies in early and/or metastatic BC.

Simulations indicate that the FRC network remains functionally resilient even when half damaged [43]. We have shown that BC metastases distort the FRC network as it invades into a node, inducing a denser, more branched topology in adjacent residual lymphoid tissue. These changes resembled those seen in uninvolved nodes, but were more accentuated. This is unlikely to result from simple diffusion of tumour‐derived soluble factors through the node as the distances are too great [44, 45]. It seems more plausible that these soluble factors are transported through the altered conduit network.

As ATB increased in all patients with BC in our cohort, survival significantly decreased. This reinforces the prognostic value of both the number and size of each nodal deposit, in both treatment‐naïve and post‐NACT cohorts. Furthermore, the extent of FRC network distortion within a metastatic deposit correlated with the size of the deposit, suggesting physical disruption as the primary driver. Once a tumour deposit exceeds a lacunarity of 12, survival worsens significantly. This could indicate a critical point at which the altered fluid flow through the FRC network triggers immunosuppression. Conversely, increased FRC alignment in tumour deposits was significantly associated with a better prognosis, which may reflect more controlled flow through the nodal parenchyma. These hypotheses need further investigation using *in vivo* and *in silico* models to elucidate how changes in FRC network architecture mechanistically influence fluid flow and immune function. We intend to use the REPLICANT model, a unique human LN perfusion system, to study fluid flow in real time and identify key molecular drivers and/or clinically relevant biomarkers [46, 47, 48].

We have shown that changes in FRC topology can be quantified using unbiased computational approaches in human ALNs with IHC, and that these changes predict prognosis in BC. By integrating this with real time flow dynamics, we are now positioned to mechanistically disentangle these complex interactions in patient‐derived samples in translationally relevant context.

## Author contributions statement

KN, SEA and AML conceived the study. AML, SLDC, CYRL, JAG, VL and DJS developed the methodology. AML undertook the investigation. AML wrote the original draft of the manuscript. KN and SEA participated in writing – review & editing. AML, KN and SEA were responsible for funding acquisition. KN, SEA, AML, SLDC and CYRL provided resources. KN and SEA supervised the study.

## Supporting information


**Figure S1.** Illustrated TWOMBLI‐derived fibroblastic reticular cell (FRC) network metrics
**Figure S2**. Differences between LN subsets driven by combined fibroblastic reticular cell (FRC) topological and clinical features
**Figure S3**. Associations between tumour infiltrated lymphocytes (TILs) and survival or clinicopathological variables in triple negative breast cancer (TNBC) and human epidermal growth factor receptor 2 (HER2)‐positive disease
**Figure S4**. Stratified analysis of fibroblastic reticular cell (FRC) network remodelling in uninvolved axillary LNs (ALNs) by triple negative breast cancer (TNBC) and neoadjuvant chemotherapy (NACT status)
**Figure S5**. Stratified analysis of fibroblastic reticular cell (FRC) network remodelling in uninvolved and residual axillary LNs (ALNs)
**Figure S6**. Clinical stratification confirms prognostic impact of molecular subtype and tumour stage
**Table S1**. Clinico‐pathological characteristics of reactive patient cohort
**Table S2**. Summary of linear regression model showing predictive power of age in reactive cohort
**Table S3**. Results of linear multivariate analysis for uninvolved nodes
**Table S4**. PCA loadings for uninvolved nodes
**Table S5**. Multivariate linear regression analysis of tumour infiltrating lymphocytes demonstrates limited association with TWOMBLI‐derived outputs in triple negative breast cancer (TNBC) and human epidermal growth factor receptor 2 (HER2)‐positive axillary LN subgroups
**Table S6**. Multivariate linear regression analysis of residual cancer burden (RCB) with other clinicopathological variables in patients with triple negative breast cancer (TNBC) and human epidermal growth factor receptor 2 (HER2) BC after neoadjuvant chemotherapy
**Table S7**. Multivariate linear regression analysis of residual cancer burden (RCB) demonstrates no association with TWOMBLI‐derived outputs in uninvolved and residual nodes from patients with triple negative breast cancer (TNBC) and human epidermal growth factor receptor 2 (HER2) BC after neoadjuvant chemotherapy
**Table S8**. Results of linear multivariate analysis for residual and uninvolved nodes
**Table S9**. Principal component analysis (PCA) loadings for residual and uninvolved nodes

## Data Availability

The data generated in this study are available within the article and its supplementary data files.
