# Supplementary material for: Topological analysis of the human lymph node reticular network predicts outcome in breast cancer
Source: J Pathol. 2026 Apr 20;269(3):348–62. doi: 10.1002/path.70065 (PMC13238348; doi:10.1002/path.70065)
Supplement: Supplementary file 1 — Figure S1. Illustrated TWOMBLI‐derived fibroblastic reticular cell (FRC) network metrics Figure S2. Differences between LN subsets driven by combined fibroblastic reticular cell (FRC) topological and clinical features Figure S3. Associations between tumour infiltrated lymphocytes (TILs) and survival or clinicopathological variables in triple negative breast cancer (TNBC) and human epidermal growth factor receptor 2 (HER2)‐positive disease Figure S4. Stratified analysis of fibroblastic reticular cell (FRC) network remodelling in uninvolved axillary LNs (ALNs) by triple negative breast cancer (TNBC) and neoadjuvant chemotherapy (NACT status) Figure S5. Stratified analysis of fibroblastic reticular cell (FRC) network remodelling in uninvolved and residual axillary LNs (ALNs) Figure S6. Clinical stratification confirms prognostic impact of molecular subtype and tumour stage Table S1. Clinico‐pathological characteristics of reactive patient cohort Table S2. Summary of linear regression model showing predictive power of age in reactive cohort Table S3. Results of linear multivariate analysis for uninvolved nodes Table S4. PCA loadings for uninvolved nodes Table S5. Multivariate linear regression analysis of tumour infiltrating lymphocytes demonstrates limited association with TWOMBLI‐derived outputs in triple negative breast cancer (TNBC) and human epidermal growth factor receptor 2 (HER2)‐positive axillary LN subgroups Table S6. Multivariate linear regression analysis of residual cancer burden (RCB) with other clinicopathological variables in patients with triple negative breast cancer (TNBC) and human epidermal growth factor receptor 2 (HER2) BC after neoadjuvant chemotherapy Table S7. Multivariate linear regression analysis of residual cancer burden (RCB) demonstrates no association with TWOMBLI‐derived outputs in uninvolved and residual nodes from patients with triple negative breast cancer (TNBC) and human epidermal growth factor receptor 2 (HER2) BC after neoadjuvan [file PATH-269-348-s001.docx]

**Topological analysis of the human lymph node reticular network predicts outcome in breast cancer**

AM Llewellyn *et al. J Pathol* <https://doi.org/10.1002/path.70065>

**Supplementary Figures S1–S6**

**Supplementary Tables S1–S9**

**Supplementary materials**

**Table of Contents**

| **Figure S1 ……………………………………………………** | **2** |
| --- | --- |
| **Figure S2 ……………………………………………………** | **4** |
| **Figure S3 ……………………………………………………** | **5** |
| **Figure S4 ……………………………………………………** | **7** |
| **Figure S5 ……………………………………………………** | **9** |
| **Figure S6 ……………………………………………………** | **11** |
| **Table S1 …………………………………………………….** | **12** |
| **Table S2 …………………………………………………….** | **12** |
| **Table S3 …………………………………………………….** | **13** |
| **Table S4……………………………………………………..** | **13** |
| **Table S5 …………………………………………………….** | **14** |
| **Table S6…………………………………………………….** | **14** |
| **Table S7 …………………………………………………….** | **14** |
| **Table S8 …………………………………………………….** | **15** |
| **Table S9 …………………………………………………….** | **15** |

**
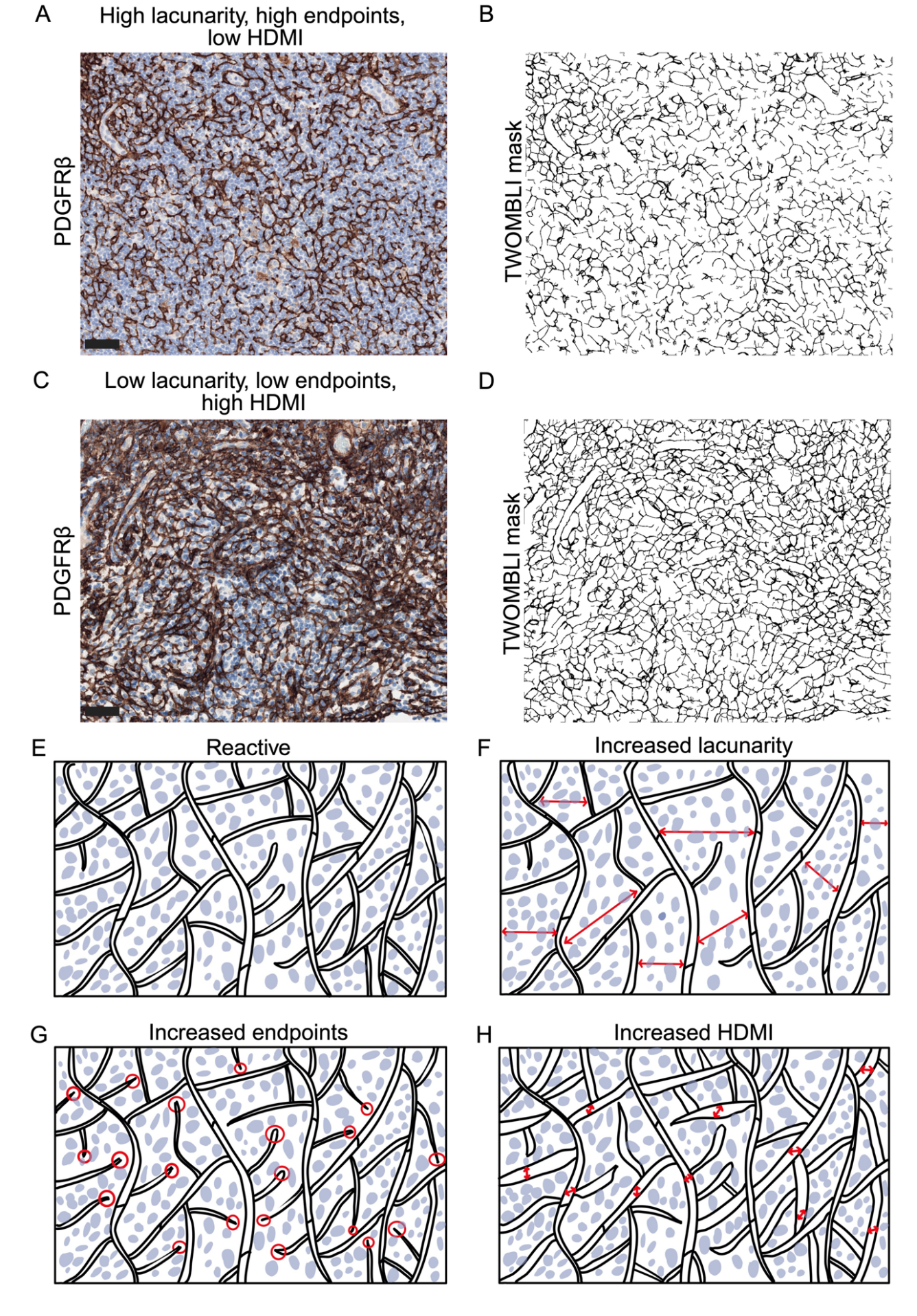
**

**Figure S1. Illustrated TWOMBLI-derived fibroblastic reticular cell (FRC) network metrics.** The TWOMBLI analysis pipeline was applied to (A, C) the platelet-derived growth factor receptor β (PDGFRβ)-stained lymph node regions of interest (ROIs) (images at ×200 magnification; scale bars, 50 µm) to generate (B, D) skeletonised representations using user-optimised parameters, from which a range of quantitative network metrics were derived. This approach enables simultaneous, unbiased assessment of multiple interconnected features of FRC network architecture and is particularly sensitive to subtle but biologically meaningful changes in network topology that are difficult to reliably assess by eye. (A, B) Representative ROIs from a reactive lymph node (lacunarity = 6.3; endpoints normalised by fibre length = 0.0186; branchpoints normalised by fibre length = 0.0631; high-density matrix intensity (HDMI) = 0.353). (C, D) Representative ROIs from an uninvolved LN from a patient with triple negative breast cancer (TNBC) (lacunarity = 3.541; endpoints normalised by fibre length = 0.0123; branchpoints normalised by fibre length = 0.0768; HDMI = 0.631). (E–H) Diagrammatic representations illustrating changes in TWOMBLI-derived parameters, with the FRC network shown in black and immune cells shown in light blue. Panel (E) represents a reactive node. Panel (F) depicts a network with increased lacunarity, characterised by a greater number and size of gaps between FRC fibres, highlighted with red arrows. Panel (G) depicts a network with increased endpoints (free ends of FRC fibres, circled in red) and reduced branchpoints (points at which multiple fibres meet), resulting in a more fragmented network. Panel (H) depicts a network with a higher HDMI, in which a greater proportion of the ROIs is occupied by the FRC network due to increased fibre width and fibre number, highlighted with red arrows.

**
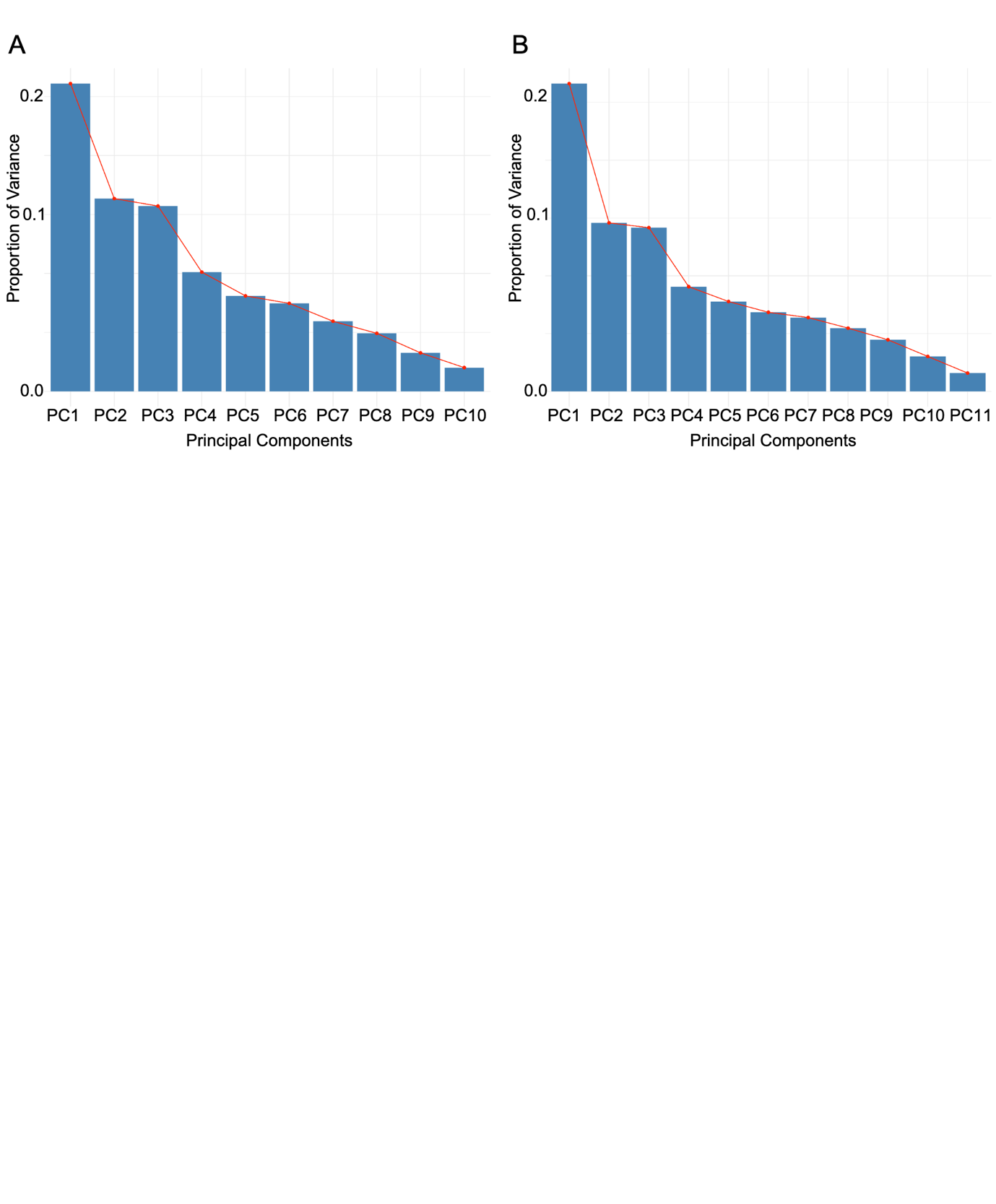
**

**Figure S2. Differences between LN subsets driven by combined fibroblastic reticular cell (FRC) topological and clinical features**. (A) Scree plot for principal component analysis (PCA) integrating TWOMBLI derived reticular network features and clinical variables from reactive, benign nodes and uninvolved LNs, shown in Figure 3. (B) Scree plot for PCA integrating TWOMBLI derived reticular network features and clinical variables from reactive, benign nodes, residual lymphoid tissue in involved nodes and uninvolved LNs, shown in Figure 4.

**
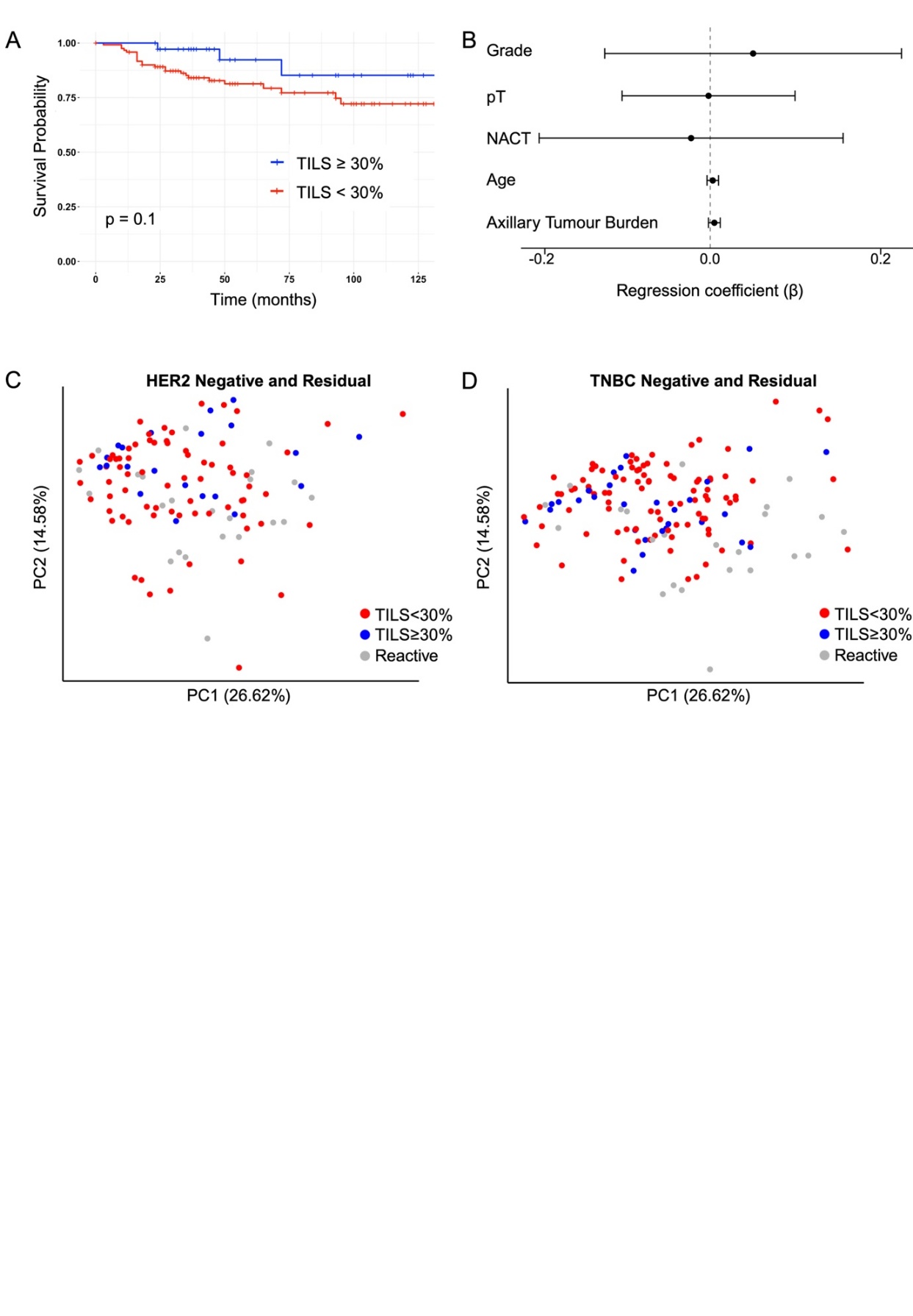
**

**Figure S3. Associations between tumour infiltrated lymphocytes (TILs) and survival or clinicopathological variables in triple negative breast cancer (TNBC) and human epidermal growth factor receptor 2 (HER2)-positive disease.** (A) Kaplan–Meier survival analysis of patients with TNBC (*n*= 87) and HER2-positive disease (*n*= 67), stratified by percentage of TILs demonstrates that those with lymphocyte-predominant BC (LPBC) had the best prognosis. However this result did not reach statistical significance, likely due to small sample size (*p*= 0.1, TILS ≥ 30%: *n*= 36, TILS < 30%: *n*= 118; Gehan–Breslow–Wilcoxon method). (B) Forest plot illustrating results of multivariate analysis performed in patients with TNBC (*n* = 87) and HER2-positive disease (*n*= 67), showing no significant correlation between LPBC and tumour grade, pathological T stage, neoadjuvant chemotherapy (NACT) exposure, age or axillary tumour burden (ATB). Forest plot shows β coefficient and 95% confidence interval. Model fit statistics: R score 0.023, F statistic 0.586, *p*= 0.7. (C, D) Principal component analysis (PCA), stratified by BC molecular subtype, was performed to integrate TWOMBLI-derived fibroblastic reticular cell (FRC) network features and clinico-pathological variables from benign control nodes (*n*= 23), uninvolved axillary LNs (LNs) and residual LN tissue from involved ALNs (shown in Figure 4), visually demonstrating lack of correlation between LPBC and other variables contributing to variance in PCA. Each data point represents the median of eight regions of interest (ROIs) per node. (C) ALNs from patients with HER2-positive disease (HER2 uninvolved: *n*= 78 nodes, HER2 residual: *n* = 15 nodes). (D) ALNs from patients with TNBC (TNBC uninvolved: *n*= 96 nodes, TNBC residual: *n*= 28 nodes).

**
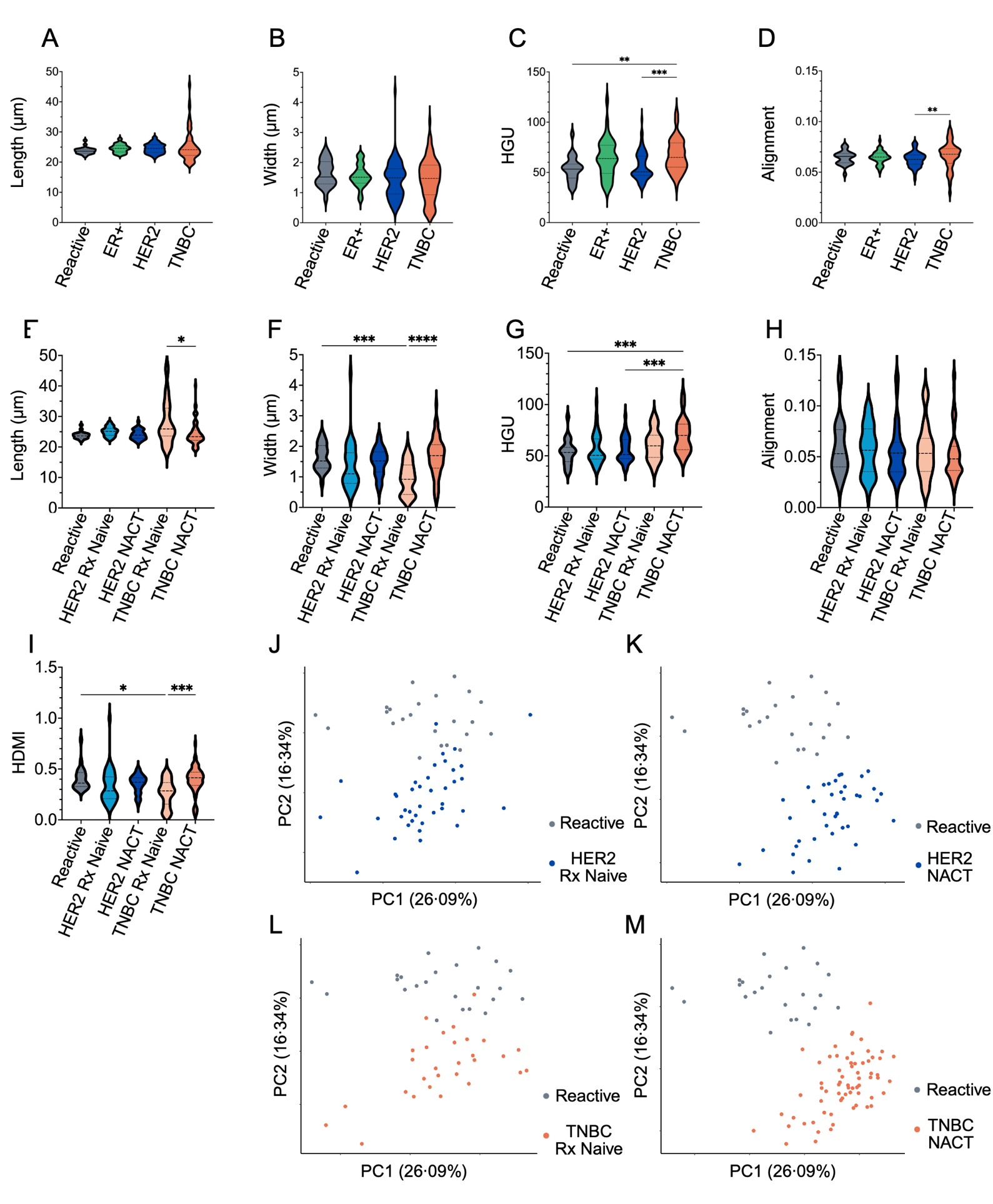
Figure S4. Stratified analysis of fibroblastic reticular cell (FRC) network remodelling in uninvolved axillary LNs (ALNs) by triple negative breast cancer (TNBC) and neoadjuvant chemotherapy (NACT status).** (A–D) Violin plots illustrating molecular subtype-specific differences in uninvolved ALN FRCs (A) fibre length (µm), (B) width (µm), (C) hyphal growth unit (HGU) and (D) fibre alignment [reactive: *n* = 23 ALNs; oestrogen receptor positive (ER+): *n*= 40 ALNs; human epidermal growth factor receptor 2 (HER2): *n*= 78 ALNs; triple negative breast cancer (TNBC): *n*= 96 ALNs]. (E–I) Violin plots showing the impact of neoadjuvant chemotherapy (NACT) on FRC (E) fibre length (µm), (F) width (µm), (G) HGU, (H) fibre alignment and (I) high-density matrix intensity (HDMI) in uninvolved ALNs, stratified by molecular subtype of BC (reactive: *n*= 23 ALNs; HER2 treatment naïve: n = 39 ALNs; HER2 post-NACT: *n*= 39 ALNs; TNBC treatment naïve: *n*= 29 ALNs; TNBC post-NACT: *n*= 67 ALNs). (J, K) Principal component analysis (PCA) comparing effects of NACT on uninvolved nodes from patients with HER2 BC (reactive: *n* = 23 ALNs; treatment naïve (J): *n*= 39 ALNs; post-NACT (K): *n*= 39 ALNs). (L, M) PCA comparing effects of NACT on uninvolved nodes from patients with TNBC (reactive: *n* = 23 ALNs; treatment naïve (L): *n* = 29 ALNs; post-NACT (M): *n*= 67 ALNs). For all graphs, each data point represents the median of eight regions of interest (ROIs) per node. All violin plots show median with interquartile range, with minimum and maximum, and were analysed using the Kruskal–Wallis test. ***p* ≤ 0.01; ****p* ≤ 0.001; *****p* ≤ 0.0001.

**
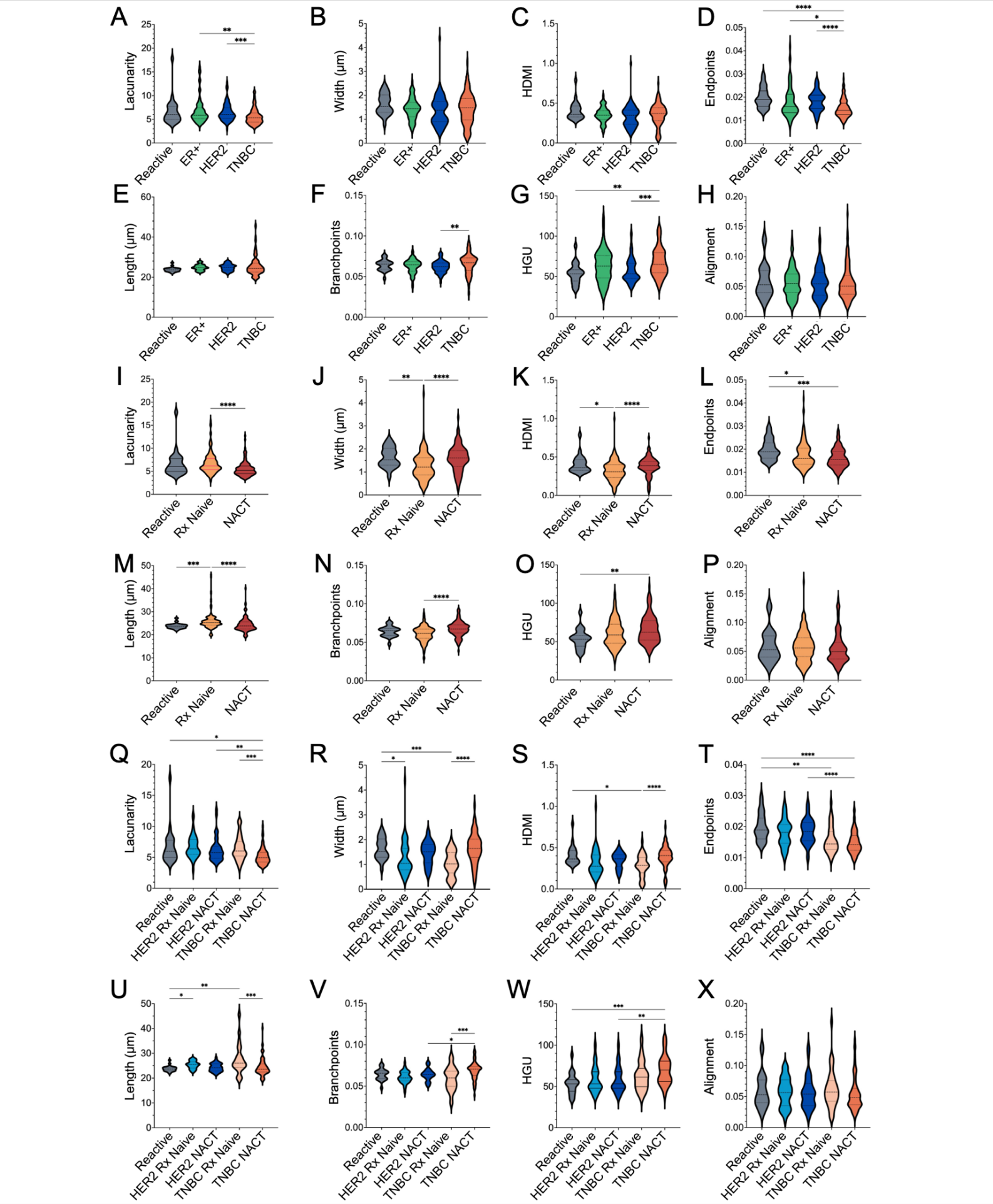
**

**Figure S5. Stratified analysis of fibroblastic reticular cell (FRC) network remodelling in uninvolved and residual axillary LNs (ALNs).** (A–X) Violin plots (median with interquartile range, with minimum and maximum) showing differences in TWOMBLI-derived parameters of uninvolved and residual ALN FRCs. (A–H) Violin plots showing molecular subtype-specific differences in (A) FRC network lacunarity, (B) fibre width (µm), (C) high-density matrix intensity (HDMI), (D) number of endpoints (normalised to fibre length), (E) fibre length (µm), (F) number of branchpoints (normalised to fibre length), (G) hyphal growth unit (HGU) and (H) alignment [reactive: *n* = 23 ALNs; oextrogen receptor positive (ER+): *n*= 69 ALNs; human epidermal growth factor receptor 2 (HER2): *n*= 93 ALNs; triple negative breast cancer (TNBC): *n*= 124 ALNs). (I–P) Violin plots showing effect of neoadjuvant chemotherapy (NACT) on (I) FRC network lacunarity, (J) fibre width (µm), (K) HDMI, (L) number of endpoints (normalised to fibre length), (M) fibre length (µm), (N) number of branchpoints (normalised to fibre length), (O) HGU and (P) alignment (reactive: n = 23 ALNs; treatment naïve: n = 150 ALNs; post-NACT = 136 ALNs). (Q–X) Violin plots stratified by exposure to NACT and molecular subtype in (Q) FRC network lacunarity, (R) fibre width (µm), (S) HDMI, (T) number of endpoints (normalised to fibre length), (U) fibre length (µm), (V) number of branchpoints (normalised to fibre length), (W) HGU and (X) alignment (reactive: *n*= 23 ALNs; HER2 treatment naïve: *n* = 49 ALNs; HER2 post-NACT: *n*= 44 ALNs; TNBC treatment naïve: *n* = 41 ALNs; TNBC post-NACT: *n* = 83 ALNs). For all plots, each data point represents the median of eight regions of interest (ROIs) per node. All graphs were analysed using the Kruskal–Wallis test. **p* ≤ 0.05; ***p* ≤ 0.01; ****p* ≤ 0.001; ****p ≤ 0.0001.

**
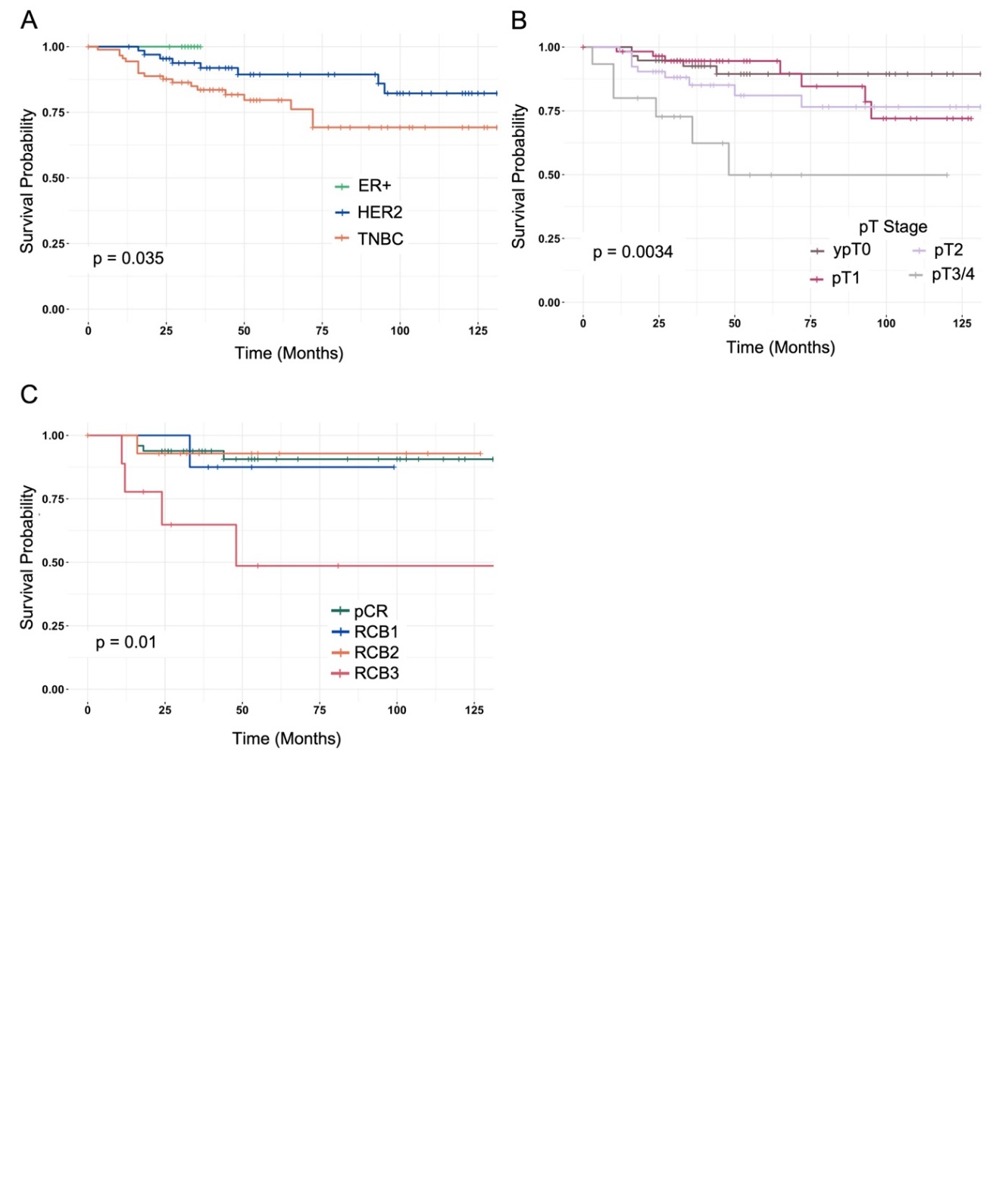
**

**Figure S6. Clinical stratification confirms prognostic impact of molecular subtype and tumour stage.** (A) Kaplan–Meier survival analysis, stratified by molecular subtype demonstrating that patients with triple negative breast cancer (TNBC) had the worst prognosis [*p* = 0.035; oestrogen receptor positive (ER+): *n* = 25 patients; human epidermal growth factor receptor 2 (HER2): *n*= 67 patients; triple negative breast cancer (TNBC): *n* = 87 patients; Gehan–Breslow–Wilcoxon method]. (B) Kaplan–Meier survival analysis stratified by pathological tumour stage (pT), showing that patients with tumours of a higher stage had worse prognosis (*p*= 0.0034; ypT0 (pathological tumour stage 0 after neoadjuvant chemotherapy): *n* = 55 patients; pT1: *n* = 59 patients; pT2: *n*= 52 patients; pT3/4: *n* = 13 patients; Gehan–Breslow–Wilcoxon method). (C) Kaplan–Meier survival analysis of patients with HER2 and TNBC exposed to neoadjuvant therapy, stratified by residual cancer burden (RCB) score, showing that patients with the most residual disease after chemotherapy (RCB3) had the worst prognosis. Note that results appear to show that patients with RCB2 had a better prognosis than those with a complete response, this is likely an artefact due to the small sample size [*p*= 0.01; pathological complete response (pCR): *n*= 52 patients; RCB1: *n*= 8 patients; RCB2: *n*= 15 patients; RCB3: *n* = 9 patients; Gehan–Breslow–Wilcoxon method].

**Table S1.** Clinico-pathological characteristics of reactive patient cohort.

| **Clinicopathological characteristic** | **No (%)** |
| --- | --- |
| **Age (years)** |  |
| 30–40 | 3 (13) |
| 40–50 | 2 (9) |
| 50–60 | 3 (13) |
| 60–70 | 4 (17) |
| 70–80 | 2 (9) |
| 80–90 | 9 (39) |
| **Site of node** |  |
| Axilla | 11 (48) |
| Cervical | 6 (26) |
| Jugular | 4 (17) |
| Upper limb (not further specified) | 2 (9) |
| **Type of biopsy** |  |
| Core biopsy | 12 (52) |
| Excisional biopsy | 11 (48) |
| **Diagnosis** |  |
| Reactive lymphoid hyperplasia | 9 (39) |
| Reactive follicular hyperplasia | 8 (35) |
| Reactive lymph node (not further specified) | 4 (17) |
| Dermatopathic lymphadenopathy | 2 (9) |

**Table S2.** Summary of linear regression model showing predictive power of age in reactive cohort.

| **TWOMBLI output** | **R Score** | **F Statistic** | ***P*-value** |
| --- | --- | --- | --- |
| **Branchpoints** | 0.10 | 2.39 | 0.137 |
| **Endpoints** | 0.15 | 3.47 | 0.076 |
| **Lacunarity** | 0.14 | 3.44 | 0.078 |
| **Alignment** | 0.03 | 0.76 | 0.394 |
| **Fibre width** | 0.06 | 1.36 | 0.257 |
| **Fibre length** | 0.06 | 1.33 | 0.262 |
| **HGU** | 0.01 | 0.22 | 0.647 |
| **HDMI** | 4.361e-06 | 9.159e-05 | 0.993 |

HDMI, high-density matrix intensity; HGU, hyphal growth unit.

**Table S3.** Results of linear multivariate analysis for uninvolved nodes.

| **TWOMBLI output** | **R Score** | **F Statistic** | ***P*-value** | **Significant predictors**  **(*p*-value)** |
| --- | --- | --- | --- | --- |
| **Endpoints (normalised)** | 0.127 | 6.748 | 0.000007 | Molecular subtype (0.0006), tumour burden in axilla (0.01) |
| **Fibre width** | 0.114 | 5.961 | 0.00003 | NACT (0.00006) |
| **Branchpoints (normalised)** | 0.109 | 5.656 | 0.00006 | NACT (0.00009), grade (0.04) |
| **Lacunarity** | 0.105 | 5.468 | 0.00009 | NACT (0.002), molecular subtype (0.02) |
| **Fibre length** | 0.090 | 4.565 | 0.0005 | NACT (0.0007), tumour burden in axilla (0.02) |
| **HDMI** | 0.088 | 4.474 | 0.0007 | NACT (0.001) |
| **HGU** | 0.073 | 3.560 | 0.004 | NACT (0.05), tumour burden in axilla (0.04) |
| **Alignment** | 0.011 | 0.517 | ns | None |

NACT, neoadjuvant chemotherapy; HDMI, high-density matrix intensity; HGU, hyphal growth unit.

**Table S4.** Principal component analysis loadings for uninvolved nodes.

| **Variable** | **PC1 Loading** | **PC2 Loading** | **PC3 Loading** |
| --- | --- | --- | --- |
| **Lacunarity** | −0.506 | −0.129 | 0.312 |
| **Branchpoints (normalised)** | 0.478 | 0.187 | −0.219 |
| **NACT** | 0.403 | −0.271 | 0.329 |
| **Endpoints (normalised)** | −0.276 | 0.285 | 0.323 |
| **Age** | −0.265 | 0.410 | −0.288 |
| **HDMI** | 0.244 | 0.343 | 0.003 |
| **Alignment** | −0.241 | −0.301 | 0.149 |
| **pT** | −0.189 | −0.147 | −0.556 |
| **Tumour burden in axilla** | −0.179 | −0.239 | −0.470 |
| **Grade** | 0.146 | −0.582 | −0.079 |

NACT, neoadjuvant chemotherapy; HDMI, high-density matrix intensity; pT, pathological tumour stage.

**Table S5.** Multivariate linear regression analysis of tumour infiltrating lymphocytes demonstrates limited association with TWOMBLI-derived outputs in triple negative breast cancer (TNBC) and human epidermal growth factor receptor 2 (HER2)-positive axillary LN subgroups.

| **Lymph node status (*n*)** | **TWOMBLI outputs included in model** | **R Score** | **F Statistic** | ***P*-value** | **Significant predictors (*p*-value)** |
| --- | --- | --- | --- | --- | --- |
| **Uninvolved nodes (174)** | HDMI, lacunarity, endpoints (normalised), branchpoints (normalised), HGU, alignment, fibre length and fibre width | 0.06 | 1.34 | 0.23 | HDMI (0.007) and fibre width (0.006) |
| **Uninvolved and residual nodes (217)** | HDMI, lacunarity, endpoints (normalised), branchpoints (normalised), HGU, alignment, fibre length and fibre width | 0.04 | 1.09 | 0.37 | HDMI (0.042) and fibre width (0.034) |
| **Metastatic nodes (50)** | HDMI, lacunarity and alignment* | 0.12 | 2.05 | 0.12 | Alignment (0.04) |

Multivariate linear regression models were constructed separately for each lymph node subgroup. R scores demonstrated limited variance explained by TWOMBLI-derived outputs across all models.

*For metastatic nodes, analysis was restricted to HDMI, lacunarity and alignment, as these were the only outputs that could be measured reliably in this subgroup.

HDMI, high-density matrix intensity; HGU, hyphal growth unit.

**Table S6.** Multivariate linear regression analysis of residual cancer burden (RCB) with other clinicopathological variables in patients with triple negative breast cancer (TNBC) and human epidermal growth factor receptor 2 (HER2) BC after neoadjuvant chemotherapy.

| **No of patients** | **TWOMBLI outputs included in model** | **R Score** | **F Statistic** | ***P*-value** | **Significant predictors (*p*-value)** |
| --- | --- | --- | --- | --- | --- |
| **84** | ATB, molecular subtype, pT, grade, TILs | 0.72 | 38.22 | < 2.2e−16 | pT (1.69e−14), ATB (0.0015), molecular subtype (0.037) |

Multivariate linear regression was performed to assess associations between RCB and other clinicopathological variables in patients with TNBC and HER2 exposed to neoadjuvant chemotherapy. The model demonstrated strong explanatory power (R = 0.72). As expected, pathological tumour stage (pT) stage, axillary tumour burden (ATB) and molecular subtype were significantly correlated with RCB, while tumour grade and tumour infiltrating lymphocytes (TILs) were not.

**Table S7.** Multivariate linear regression analysis of residual cancer burden (RCB) demonstrates no association with TWOMBLI-derived outputs in uninvolved and residual nodes from patients with triple negative breast cancer (TNBC) and human epidermal growth factor receptor 2 (HER2) BC after neoadjuvant chemotherapy (NACT).

| **Lymph node status (n)** | **TWOMBLI outputs included in model** | **R Score** | **F Statistic** | ***P*-value** | **Significant predictors (*p*-value)** |
| --- | --- | --- | --- | --- | --- |
| **Uninvolved and residual nodes (127)** | HDMI, lacunarity, endpoints (normalised), HGU, branchpoints (normalised), alignment, fibre length, fibre width | 0.11 | 1.60 | 0.13 | None |

A multivariate linear regression model was constructed to evaluate associations between RCB and TWOMBLI-derived outputs in uninvolved and residual nodes from patients with TNBC and HER2 exposed to NACT. The model demonstrated limited explanatory power (R = 0.11), and no TWOMBLI-derived variables were independently associated with RCB. HDMI, high-density matrix intensity; HGU, hyphal growth unit.

**Table S8.** Results of linear multivariate analysis for residual and uninvolved nodes.

| **TWOMBLI output** | **R Score** | **F Statistic** | ***P*-value** | **Significant predictors (*p*-value)** |
| --- | --- | --- | --- | --- |
| **Lacunarity** | 0.157 | 9.343 | 0.000000002 | Size of met (0.00001), NACT (0.001), molecular subtype (0.008) |
| **Fibre width** | 0.125 | 7.179 | 0.0000004 | NACT (0.00002), size of met (0.04) |
| **HDMI** | 0.122 | 5.210 | 0.00004 | NACT (0.001) |
| **Endpoints (normalised)** | 0.112 | 6.374 | 0.0000002 | Molecular subtype (0.0006), tumour burden in axilla (0.004) |
| **Fibre length** | 0.092 | 5.087 | 0.00005 | NACT (0.00009), tumour burden in axilla (0.02), molecular subtype (0.04) |
| **Branchpoints (normalised)** | 0.010 | 6.862 | 0.0000008 | NACT (0.00002), size of met (0.05) |
| **HGU** | 0.072 | 3.895 | 0.0009 | Tumour burden in axilla (0.01), NACT (0.02) |
| **Alignment** | 0.026 | 1.127 | ns | Size of met (0.03) |

HDMI, high-density matrix intensity; HGU, hyphal growth unit; NACT, neoadjuvant chemotherapy.

**Table S9.** Principal component analysis (PCA) loadings for residual and uninvolved nodes.

| **Variable** | **PC1 Loading** | **PC2 Loading** | **PC3 Loading** |
| --- | --- | --- | --- |
| **Lacunarity** | 0.410 | 0.256 | −0.124 |
| **Branchpoints (normalised)** | −0.467 | −0.182 | 0.154 |
| **Endpoints (normalised)** | 0.336 | 0.416 | 0.104 |
| **NACT** | −0.336 | 0.218 | −0.421 |
| **HDMI** | −0.312 | 0.024 | 0.264 |
| **Size of metastasis** | 0.234 | −0.286 | −0.161 |
| **pT** | 0.219 | −0.516 | −0.014 |
| **Alignment** | 0.208 | 0.025 | −0.234 |
| **Age** | 0.176 | −0.162 | 0.542 |
| **Tumour burden in axilla** | 0.162 | −0.516 | −0.062 |
| **Grade** | −0.077 | −0.194 | −0.569 |

HDMI, high-density matrix intensity; NACT, neoadjuvant chemotherapy; pT, pathological tumour stage.
